# Bridging the gap of axonal regeneration in the central nervous system: A state of the art review on central axonal regeneration

**DOI:** 10.3389/fnins.2022.1003145

**Published:** 2022-11-09

**Authors:** Gonçalo Costa, Filipa F. Ribeiro, Ana M. Sebastião, Elizabeth M. Muir, Sandra H. Vaz

**Affiliations:** ^1^Instituto de Medicina Molecular João Lobo Antunes, Faculdade de Medicina, Universidade de Lisboa, Lisbon, Portugal; ^2^Instituto de Farmacologia e Neurociências, Faculdade de Medicina, Universidade de Lisboa, Lisbon, Portugal; ^3^Faculdade de Medicina, Universidade do Porto, Porto, Portugal; ^4^Department of Physiology, Development and Neuroscience, University of Cambridge, Cambridge, United Kingdom

**Keywords:** neuronal regeneration, central axonal regeneration, glial scar, stem cells, neurodegeneration, central nervous system injury

## Abstract

Neuronal regeneration in the central nervous system (CNS) is an important field of research with relevance to all types of neuronal injuries, including neurodegenerative diseases. The glial scar is a result of the astrocyte response to CNS injury. It is made up of many components creating a complex environment in which astrocytes play various key roles. The glial scar is heterogeneous, diverse and its composition depends upon the injury type and location. The heterogeneity of the glial scar observed in different situations of CNS damage and the consequent implications for axon regeneration have not been reviewed in depth. The gap in this knowledge will be addressed in this review which will also focus on our current understanding of central axonal regeneration and the molecular mechanisms involved. The multifactorial context of CNS regeneration is discussed, and we review newly identified roles for components previously thought to solely play an inhibitory role in central regeneration: astrocytes and p75NTR and discuss their potential and relevance for deciding therapeutic interventions. The article ends with a comprehensive review of promising new therapeutic targets identified for axonal regeneration in CNS and a discussion of novel ways of looking at therapeutic interventions for several brain diseases and injuries.

## Introduction

The human brain harbors an array of unique capabilities not found anywhere else in the animal kingdom. It is undoubtedly the most advanced brain known of any living being. Thus, it is interesting to ponder why some capabilities intrinsic to less evolved animals that seem clearly beneficial for survival, such as regeneration, appear to be dramatically reduced in the human central nervous system (CNS) (Anderson et al., 2016, 2018; Adams and Gallo, 2018; Fawcett, 2020; Yang et al., 2020).

Neurodegenerative diseases (ND), stroke, spinal cord injury (SCI) and traumatic brain injury (TBI) are just few examples for which it seems clear that, evolutionarily speaking, it would make sense to have retained some CNS regenerative capacity that simpler life forms possess (e.g., teleost fish, axolotls). Upon injury, not only does the human CNS create a non-permissive environment for axonal regeneration through the formation of the glial scar (Anderson et al., 2016; Adams and Gallo, 2018; Wang H. et al., 2018), but also CNS neurons, in contrast to peripheral nervous system (PNS) neurons, possess intrinsic characteristics that drastically limit axonal regeneration. For instance, cargo and protein transport, regenerative capacity, microtubule density and axon diameter are all less pronounced in the CNS compared to the PNS (Reier et al., 2017; Fawcett, 2020). Thus there are many studies documenting robust axonal regeneration in the PNS (Wang et al., 2009; Ding et al., 2010; Xu et al., 2012; Zhang et al., 2021) but very few in the CNS.

Nonetheless, studies attempting to restore function and structure to severed axonal processes in the CNS have been increasing over the years and it is clear there is a need for the development of such a therapy, given the broad spectrum of brain-related injuries and diseases that it could potentially impact on. Indeed, this topic has already been addressed by others (Silver et al., 2015; Tran et al., 2021), as having a multifactorial nature which has been acknowledged for many years. With this in mind, the purpose of this review is to shine a light on the current knowledge regarding the molecular mechanisms and the cellular and cytoskeleton dynamics at play following CNS injuries. We will discuss the key factors impeding axonal growth after injury, take this information and compile works done so far that have shown promise in eliciting CNS axonal regeneration, while describing the most common ways to search for new methods and therapeutic interventions. We finally discuss what research in this area should focus on in order to optimize therapeutic attempts to maximize axonal regeneration capacity in the CNS.

## Peripheral nervous system versus central nervous system regeneration: The required starting point

In order to gain insights into what is at play when we talk about axonal regeneration in the context of the CNS, first, one must have a clear picture of what regeneration of axonal processes involves in a context where its potential is higher and extensive regeneration is possible – the PNS.

### Peripheral nervous system mechanism for regeneration

In the PNS nerve regeneration is widely observed. This can largely be attributed to the multitude of roles played by myelinating and non-myelinating Schwann cells. These cells are a driver and guiding force during development for axons of the periphery. Upon injury, Schwann cells undergo plasticity events that allow them not only to demyelinate the severed ends of an injured axon but also to potentiate regeneration by upregulating various pro-regenerative related genes and transcriptional mechanisms [e.g., mitogen-activated protein kinase, sonic hedgehog (Shh)] (Nocera and Jacob, 2020).

**FIGURE 1:**
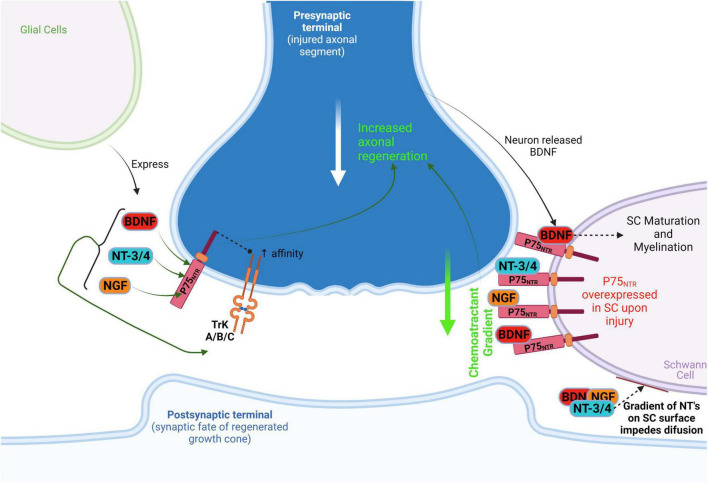
Schwann cellprotect (SC) action on PNS regenerative axonal guidance: the role of P75_NTR_. After injury, SC overexpresses P75_NTR_ which acts as a neurotrophin receptor and as a booster of neurotrophin (NGF, BDNF, NT-3/4) affinity to TrK(A/B/C). In addition, the P75_NTR_ is suggested to act as a presenting molecule at SC surface for autocrine neurotrophins in degenerating/injured neurons, thus preventing diffusion of these neurotrophins on SC and generating a chemoattractant gradient that guides/attracts axons. This process leads to axonal regeneration toward non-injured distal neurons. Neuronal released BDNF acts on SC P75_NTR_ to promote SC maturation and myelination, perpetuating this chemoattractant gradient as well. BDNF, brain-derived neurotrophic factor; NT-3/4, neurotrophins 3 and 4; NGF, nerve growth factor.

This is relevant because, apart from the reprograming that Schwann cells experience upon injury, they are capable of digesting myelin debris derived from injured axons and guide these severed processes through a non-permissive environment. Schwann cells transplanted into CNS injuries facilitate axon regeneration, although to a lesser extent than in the PNS because the extracellular space of injury in the CNS contains inhibitory components like myelin associated growth inhibiting proteins released by cells such as oligodendrocytes and the chondroitin sulfate proteoglycans (CSPGs). Additionally, repulsive axon guidance signals are expressed (e.g., netrins, intrins, semaphorins) which make it a much less axonal growth permissive environment than in the PNS (Schaffar et al., 2008; Duraikannu et al., 2019; Uyeda and Muramatsu, 2020). Nevertheless, Schwann cells can act as the bridge between the injured axonal processes promoting growth cone formation and migration (Gordon et al., 2009). It seems they act through the p75 neurotrophin receptor (p75NTR) to act as these axonal guiding forces for regenerating PNS neurons (Bentley and Lee, 2000), something to be further discussed ahead in the context of CNS regeneration (Figure 1).

Schwann cells are not solely responsible for PNS regeneration, because there are other factors involved, but they do provide a bridge to guide axonal regeneration in the PNS. Also, Schwann cells behave in a stem cell-like manner, in the sense that they undergo a clear reprograming event, and through a combination of extracellular matrix (ECM) debris clearance and epigenetic alterations, act as the main facilitators of regeneration in the PNS (Reier et al., 2017; Duraikannu et al., 2019; Nocera and Jacob, 2020).

A comparison of the response of central and peripheral branches of DRGs to axotomy also gives valuable insights into the mechanisms required to mount a successful regenerative response. Kong et al. (2020) used this approach to conduct proteomics on axoplasm enriched samples derived injured PNS or CNS. They demonstrated that AMPKα1 was downregulated following PNS but not CNS injury and that this was a result of degradation via 26S proteasome (Kong et al., 2020). The 19S regulatory subunit PSMC5 is required for proteosome assembly and activation and in turn it is phosphorylated and activated by CAMKIIα. Immunostaining revealed that AMPK was predominantly expressed by mechanoceptive and proprioceptive neurons of the DRGs. Moreover, AMPKα1 inhibition as well as deletion enhanced regeneration of DRG neurons *in vitro* on both permissive and inhibitory substrates and was accompanied by activation of multiple regenerative signaling pathways implicating an important role for AMPK in regulating regeneration. The study also showed that the findings were replicated *in vivo.* Conditional deletion of AMPK prior to a dorsal column crush resulted in robust regeneration of sensory axons that entered and crossed the injury site. The results are promising and identify a new target for SCI therapy. However, the effects maybe specific to sensory neurons as AMPK activation via LKB1 produces a regenerative response in cortical neurons, suggesting distinct signaling pathways may be employed by different neuronal subtypes.

In another study, the epigenetic events that follow PNS and CNS injury were investigated. Using RNAseq, ATAC, and ChIPseq to analyze epigenetic changes occurring following sciatic nerve axotomy (SNA) and dorsal column axotomy (DCA), it was reported that significant changes in epigenetic signature follow SNA but that the changes following DCA were different and more modest (Palmisano et al., 2019). They observed that following SNA, H3K9ac and H3K27ac, which are associated with active chromatin, were enriched at the transcription start sites (TSS) and gene bodies of a unique set of genes and that this was accompanied by enhanced chromatin accessibility as assessed by ATAC which uses a topoisomerase to insert sequencing primers in areas of accessible chromatin. Thus, SNA promotes the formation of more relaxed chromatin, which correlates with enhanced gene expression. Differentially expressed genes following SNA include those associated with regenerative signaling pathways such as JAK/STAT and mTOR pathways. In contrast, the differentially expressed pathways activated following DCA were those associated with neurological disorders and mitochondrial oxidative phosphorylation. Interestingly, whilst application a histone deacetylase inhibitor resulted in an increase in histone acetylation and chromatin accessibility, the repertoire of differentially expressed genes had only a 12% overlap with those that follow SNA, suggesting that additional mechanisms are at play following SNA. They also identify the chromatin organizer CTCF as an important factor regulating chromatin topology during the regenerative response. CTCF (CCCTC binding factor) binding sites were identified in many promoters and enhancers of injury- induced genes and the conditional deletion of CTCF resulted in delayed regeneration following sciatic nerve crush (SNC). The authors point out however, that not all of the promoters and enhancers of differentially expressed genes are occupied by acetylated histones or are located in regions of accessible chromatin. This suggests additional mechanisms are employed to mark the promoters and enhancers of active genes.

In a different investigation Sahoo et al. (2018) use DRG neurons to study translation of stored mRNAs in the axons of these neurons in different growth states. They measured protein synthesis levels in the axonal compartment of naïve neurons and compare them with those in the axons of neurons that have undergone injury. They show that in the axons of naïve neurons, translation is negatively regulated by the Ras GAP SH3 domain binding protein (G3BP1) which predominantly associates with stress granule (SGs)-like structures in the axon. In naïve neurons GABP1 exists in an aggregated form bound to SGs, but upon regeneration these structures disperse as do the GBP1 aggregates. This is a result of G3BP1 phosphorylation which blocks oligomerization of GBP1. This dispersal results release of the mRNAs bound to these structures and their local translation. They use three different mRNAs to demonstrate the existence of distinct regulatory mechanisms for controlling translation in the axon and show that these are related to the growth status of the axon. They studied the GAP-43, Importin-β (Imp-β) and Neuritin 1 (Nrn-1). Using immunoprecipitation they demonstrate an association of GBP-1 with Imp-β and Nrn-1 but not GAP-43 mRNAs in naïve neurons. In DRGs which had been subject to a conditioning lesion Imp-β showed more association with GBP-1 and the opposite was found for Nrn-1. This makes sense as the continuous expression of Imp-β would likely decrease axon elongation due to its role in axon length sensing. Therefore axon growth after a conditioning injury could be aided by sequestering it’s translation. In contrast Nrn-1 promotes regeneration, thus it’s continued translation would be beneficial to regeneration. Taken together these experiments show that SGs are involved in the regulation of translation of some but not all mRNAs in the axon and as expected overexpression of GBP-1 decreased translation of Imp-β and Nrn-1 but not GAP-43. In an extension of this study the roles of the different domains of the GBP-1 protein were assessed. Expression of the different domains of GBP-1 revealed the acidic B domain promoted neurite outgrowth in cultures of naïve DRG neurons. The same constructs were also assessed *in vivo* following SNC with similar findings. Expression of the B domain resulted in significantly enhanced regeneration and this resulted in accelerated functional recovery as assessed by measurement of compound muscle action potentials. Immunoprecipitation studies showed that less of the Nrn-1 and Imp-β mRNAs co-precipitated with full length GBP-1 when the B domain was expressed, consistent with expression of this domain causing release of these mRNAs from the GBP-1. Indeed, transduction of constructs encoding the full length B domain or a peptide composed of amino acids 190–208 resulted in enhanced protein synthesis likely a result of disruption of GBP-1 function by the release of specific mRNAs. Interestingly, whilst Nrn-1 protein levels were increased the levels of Imp-β were not, suggesting that translation of Imp-β requires an additional signal. In summary, the authors have demonstrated a role for GBP-1 in the regulation of mRNA translation in DRG neurons. Moreover, they have shown that a fragment of the B domain of GBP-1 enhances protein synthesis and axon-outgrowth and thus is a potential therapeutic candidate for promoting regeneration in the CNS.

The majority of previous studies on DRGs have used whole DRGs which has several drawbacks. First, they are composed of many cell types both neuronal and non-neuronal. Second, there are at least nine different neuronal subtypes present which perform different functions for example proprioceptors, nociceptors and pruriceptors. Lastly, not all the axons of the neurons present are axotimized following injury. A further complication is that cell dissociation procedures can act as injury signals. Renthal et al. (2020), solve these problems by using the nuclei isolated from single cells for their analysis. They perform RNAseq on these nuclei and identify the changes in gene expression that take place following injury. They used three different injury models performed on mouse DRGs isolated from the lumbar region of the spinal cord. Spinal nerve transection spNT, sciatic nerve transection ligation ScNT and Sciatic nerve crush ScNC. Interestingly, only ScNC results in full axonal regeneration and target re innervation but this allows gene expression analysis at the later stages of regeneration and repair. They report that the injury response initiates 3–7 days post-injury (PI) and that small diameter neurons initiated transcriptional changes earlier than large diameter neurons. Although some neuronal cells types responded with unique changes in gene expression the majority of the changes that occurred were common across all neuronal cell types and included previously documented RAGs such as ATF-3, Jun, Sox11 and genes affecting excitability including those encoding neuropeptides and ion channels. Intriguingly, the upregulation of these RAGs was accompanied by the downregulation of genes which specify neuronal cell type. The changes in the non-neuronal cell types exhibited smaller changes and the genes upregulated were related to axon guidance regeneration and pain. Moreover, similar changes were seen across all injury models and in both proximal and distal injuries. Using, ScNC injury model the authors show that these changes are reversed following completion of regeneration and reinnervation with cell type specific gene expression regained as the neurons return to their naïve state.

These studies were extended to identify the key drivers of the injury response. One day PI, several genes are upregulated including SOX11, ATF3, JUN, KLF6, KLF7, SMAD1, ETS2, and Bhlhe41. Of these ATF3 was the mostly highly expressed and its binding sites were most enriched in the induced RAGs. It was therefore selected for further study using ATF3 KO mice. They showed ATF3 KO mice exhibited delayed recovery after nerve crush and this correlated with lower expression of injury-associated genes at 1.5 and 7 days PI. Interestingly, the down regulation of the cell type specific genes was also attenuated in these mice suggesting an additional role for ATF-3 in this process. However, the authors suggest that this may be an indirect effect as there are no canonical ATF-3 binding sites present in the genes that determine neuronal specificity. It is of note that overexpression of ATF-3 in the CNS promotes regeneration and the hypothesis that regeneration may require expression of developmentally regulated TFs is not corroborated by this study.

In a follow up study Cheng and collaborators investigate the regulation of ATF-3 in more detail (Cheng et al., 2021). There are many mechanisms that regulate the induction of RAG genes following PNS injury. A Ca^2 +^ wave that follows axotomy activates PKC which results in export of HDAC5 from the nucleus with consequent increase in histone acetylation and activation of a pro-regenerative program. ATF-3 is de methylated. In this study an additional method of ATF-3 activation is identified. They show that DNA breaks at the ATF-3 locus caused by DNA Topoisomerase I induce activation of ATF-3 and consequent induction of RAG gene expression. Moreover, they demonstrate that this effect can be accelerated by application of camptothecin, a topoisomerase1 inhibitor which results in the accumulation of more double stand DNA breaks as it prevents the religation of the nicked DNA. Injections of camptothecin resulted in accelerated expression of RAGs early after injury (18–24 h) which returned to normal compared to controls by 36 h. This was accompanied by increased neurite outgrowth of sensory neurons *in vitro* and enhanced regeneration following SNC *in vivo*. Thus, this topoisomerase inhibitor mimics the effect of a preconditioning lesion and is thus a potential therapeutic candidate for promoting PNS repair where a preconditioning lesion is not clinically applicable.

Collectively, these studies have significantly advanced our knowledge of the mechanisms that take place when PNS neurons mount a regenerative response and have identified TFs and chromatin remodeling as key regulators of the process. These studies and others also give insights into the major barriers to regeneration (Mahar and Cavalli, 2018). This will aid in the development of therapies designed to promote regeneration in the CNS. Indeed, in DRG neurons activation of the transcription factor (TF) Jun occurs in response to injury and is required for PNS regeneration. However, such activation of Jun is not observed following CNS injuries and overexpression of Jun in cultured cortical neurons enabled them to regenerate. ATF3 is similarly differentially expressed in the PNS and CNS following injury and overexpression of ATF3 in cortical neurons also promoted regeneration. It is of note that whilst overexpression of a combination of Jun and ATF3 enhanced regeneration of DRG neurons they did not act in a synergistic manner in cortical neurons highlighting the fact that subtle differences between exist between neuronal subtypes. It is of note here, that studies on cortical neurons showed that chromatin accessibility declined with age during development which may provide an intrinsic barrier to regeneration as the pro-regenerative TFs may be blocked from binding to the promoters and enhancers of pro-regenerative genes. However, the studies described herein also underscore the fact that regenerative mechanisms observed in the PNS will not necessarily be recapitulated in the CNS. For example, the dual leucine zipper kinase (DLK) an early sensor of injury in the PNS, is activated by microtubule instability and by the rises in cAMP levels that are induced by injury. In the PNS DLK is important for activation of several pro-regenerative pathways however, its pro-regenerative role cannot be assumed for all injury paradigms as it’s activation in RGCs results in cell death. These findings emphasize the different transcriptional responses to injury between neuronal subtypes and imply the use of different gene regulatory networks to control regeneration and cell death. Furthermore, outcomes need to be interpreted with caution as exampled by the observation that SOX11, which is one of the RAGs associated with regeneration in the PNS, promotes regeneration of the CST following SCI but interferes with functional recovery.

In a very recent study Cheng et al. (2022) use integrative genomics and bioinformatics approaches to probe data sets of genes differentially expressed following injury to the PNS and CNS (Cheng et al., 2022). They identify the RE1 silencing factor REST as an upstream silencer of the regenerative program. These algorithms were used to probe data sets obtained from many PNS injury models and uncovered a subnetwork of interconnected TFs which were conserved across all the injury paradigms namely Jun, Stat3, Sox11, Smad1 and Atf3. These were not induced or only transiently induced following CNS injury. Importantly, they show that REST negatively regulates the expression and interaction of these TFs acting as a master switch to turn off these genes which would normally drive regeneration. They validate their approach for identifying genes important for CNS regeneration by showing that deletion of REST or manipulation of it’s expression with the use of a dominant negative resulted in RAG gene expression and enhanced neurite outgrowth both *in vitro* and *in vivo*. This was demonstrated in two CNS injury models, optic nerve crush and SCI. Moreover, these negative effects of REST were only observed in the context of injury as deletion of REST had no effect in control uninjured animals or on DRGs plated on laminin as opposed to CSPGs. Thus the authors have shown that integrated genomics and bioinformatics can be a powerful method of identifying genes important for CNS regeneration. Uncovering a key master switch that regulates the expression of core RAGs is huge step forward in advancing our knowledge about how to boost the intrinsic ability of CNS neurons to regenerate.

### Central nervous system: The lack of a bridge to enable neurons to cross the scar

These beneficial regenerating roles of PNS Schwann cells are not present during CNS regeneration. In the CNS, apart from a lower capacity for protein transport, regeneration, microtubule density and axon diameter (Reier et al., 2017; Fawcett, 2020), the key event that limits regeneration is the formation of the highly inhibitory glial scar that forms following injury (Karova et al., 2019; Yang et al., 2020). Moreover, the myelinating cells of the CNS are oligodendrocytes which in contrast to Schwann cells, are not pro-regenerative. Factors and debris released following their death or damage together with proteins expressed on their cell surface (e.g., Neurite outgrowth inhibitor – NogoA, Myelin associated glycoprotein – MAG, Oligodendrocyte myelin glycoprotein -OMgp) are highly inhibitory for the central regenerative machinery (Dickendesher et al., 2012; Yeung et al., 2014). This further supports the idea that a requirement for CNS regeneration is axonal guidance beyond this scar, involving not only removal of the cellular debris but also activation of transcriptional pathways, which promote regeneration in the CNS in a similar way to the mechanisms, which take place in the PNS. An additional requirement will be removal of the major inhibitory components present, such as the chondroitin sulphate proteoglycans (CSPGs).

## The glial scar: Its impact and cellular players involved

After injury to the CNS, be it traumatic, degenerative or ischemic, an anti-regenerative environment develops (Anderson et al., 2016; Adams and Gallo, 2018; Yang et al., 2020). This consists of a glial scar which is rich in molecules highly inhibitory to axonal regrowth. There is also a robust cellular response to the injury (Wanner et al., 2013; Anderson et al., 2016; Adams and Gallo, 2018; Yang et al., 2020). Although it is important to note that there are differences in scar composition and size depending on the injury location and on the injury type (Adams and Gallo, 2018), there are players and characteristics that they all have in common (Wang H. et al., 2018). We will describe them in chronological order with respect to the time of injury mentioning, when relevant, key known differences between injury types and how that might impact on more generally applicable therapeutic targeting.

### Formation and timeline of the glial scar

**FIGURE 2:**
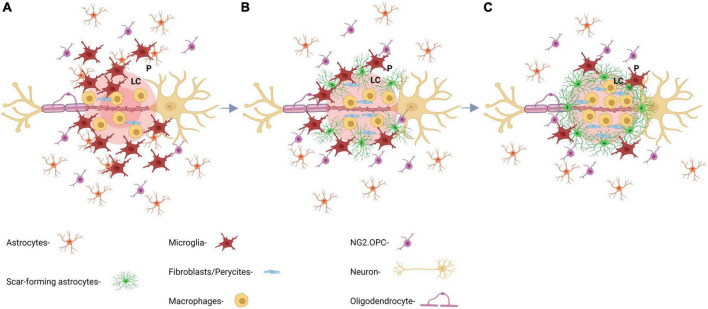
Key aspects of the formation and maturation process of the glial scar. **(A)** At day 1 post-injury (dpi), there is an initial recruitment of reactive astrocytes by inflammatory microglia and macrophages releasing cytokines. Astrocytic processes are extended perpendicularly toward the lesion core. **(B)** At 7 dpi, astrocytic processes start to intertwine with each other making up the barrier region of the scar and forming a border between ECM and glial scar, which prevents further inflammation propagation. **(C)** At 14 dpi, the glial scar is considered mature and starts to shrink. GFAP expression decreases drastically after this time point and ECM continues to overexpress CSPG.

The formation of a mature glial scar after injury can be described as a sequence of three main events (Figure 2). First, as the first neuroinflammatory response, microglia, macrophages, and nerve/glial antigen 2 (NG2+) glia are recruited to the lesion. Then, in a second phase, astrocytes, mostly activated by microglia-released cytokines, become reactive or differentiate into reactive astrocytes from NG2+ precursors. Reactive astrocytes with hypertrophied morphology, migrate to the penumbra and extend their processes to the lesion core. Finally, at around the 2nd week, the scar matures and the astrocyte processes become parallel to the lesion core and intertwine with each other forming the mature penumbra and the defined borders of the lesion (Wang H. et al., 2018; Yang et al., 2020).

Reactive astrocytes are key cellular players in the context of CNS injury, therefore its correct identification is essential. Although GFAP is a very used marker for astrocytes, there is accumulating evidence that GFAP expression may not serve as a sole marker of astrocyte reactivity as other cell types express considerable amounts of GFAP. GFAP overexpression by astrocytes does not entirely correlate with a specific reactive phenotype (Anderson et al., 2016; Escartin et al., 2021). A combination of hypertrophy of astrocytic processes along with other markers (e.g., Aldh1/GFAP) might serve as a more accurate method to identify the state of astrocyte reactivity (Escartin et al., 2021). Moreover, the population of reactive astrocytes present at the lesion site following SCI have been shown to be heterogeneous. For example only a subset of express the neural stem cell markers nestin and SOX-2 and their phenotype can differ with distance from the lesion site for example astrocytes present in the SCI scar, upregulate GFAP, become hypertrophic, change orientation, become dense and block regeneration whilst astrocytes far from the lesion site upregulate GFAP, don’t re-orientate and support regeneration (Yang et al., 2020).

After glial scar maturation, at around the 2nd week post-injury, GFAP expression was described to decrease rapidly after a hippocampal stab wound injury (HSI) (Zhu et al., 2003). This is interesting because such a steep decline in GFAP expression is not observed in other injury models, for example SCI models. It is of note that the extent of astrocyte activation is stronger post SCI compared to TBI, which illustrates differences between injury types.

The previous point illustrates the heterogeneity of the glial scar, which depends on injury type and location and emphasizes the fact that one cannot make general assumptions that all glial scars will form and behave in the same way with regard to the extent to which they inhibit axon outgrowth and regeneration. However, the important role that astrocytes play following injury to the CNS seems to be common and undebatable.

### Lesion core and penumbra–Cellular players

The roles of the glial scar following CNS injury are controversial. One school of thought is that: (i) it acts as a physical barrier for regenerating axons (for a review see: Gallo and Deneen, 2014; Wang H. et al., 2018; D’Ambrosi and Apolloni, 2020); and (ii) it consists of an extracellular matrix (ECM) containing highly inhibitory molecules for neuronal regeneration that are overexpressed upon injury (Daniell, 2012; Yang et al., 2020). These include a class of molecules called the CSPGs such as brevican, neurocan, phosphacan, versican, aggrecan, which inhibitory to axon regeneration (Zhao et al., 2011; Daniell, 2012; Gallo and Deneen, 2014; Wang H. et al., 2018; D’Ambrosi and Apolloni, 2020; Sami et al., 2020; Yang et al., 2020). Furthermore, activated macrophages which infiltrate the lesion have been shown to be responsible for the observed axon die back which occurs following injury. These dystrophic axon bulbs then bind to CSPGs on the surface of NG2 + OPCs via the CSPG receptor PTPσ, forming a synapse-like structure where they remain indefinitely unable to regenerate. It is suggested that this maybe a response to limit damage from activated macrophages (Silver, 2016). The other school of thought is that the scar is not an absolute barrier to regeneration and further that enhanced regeneration can be achieved by supplying trophic factors and by boosting the intrinsic capacity of the neurons to regenerate in order to stimulate the reorganization of spared circuits. For example spared propriospinal circuits can reorganize to form detour circuits which connect either side of the lesion site. Thus, long distance regeneration is not required for functional recovery, however, combining such strategies with some form of rehabilitation such as electrical stimulation is essential. Evidence is also presented which supports the view that strategies that remove CSPGs or neutralize myelin proteins act primarily by stimulating plasticity rather than promoting regeneration *per se* (Sofroniew, 2018).

However, in the last decade multiple reports have emerged warning caution when attributing an exclusively inhibitory role to the glial scar in axonal regeneration (Zhao et al., 2011; Yao, 2018; Yang et al., 2020). With respect to the beneficial roles of the glial scar there is strong evidence that it plays multiple and key beneficial functions that contribute to a possible rescue of injured axons: (i) the physical barrier property of the glial scar prevents lesion/inflammatory propagation and macrophage spread beyond the lesion core (Anderson et al., 2018; Yang et al., 2020); microglia form a barrier to infiltrating leukocytes and to astrocytes which have become activated in response to microglial-derived IGF-1 and astrocytes block off infiltrating fibroblasts (Tran et al., 2021). Indeed, selective ablation of microglia results in a reduction in scar formation but results in worse outcomes (Yang et al., 2020). Thus, after the acute stage of SCI the glial scar acts as a restrictive boarder to limit the formation of fibrotic tissue and macrophage infiltration. Interestingly, delayed removal of the chronic scar 5 weeks PI didn’t improve regeneration which suggests fibrotic tissues and macrophages in the lesion core are still active and detrimental in the chronic scar (Yang et al., 2020). (ii) The glial scar also balances inflammatory activity occurring in response to injury. Reactive astrocytes not only limit inflammation but also contribute to it (Didangelos et al., 2014; Wang H. et al., 2018); (iii) astrocytes in stem cell-like states have been reported to form bridges across the lesion core allowing for axonal crossing and regeneration. This phenomenon, exclusive to immature astrocytes, provides a microenvironment which favors axon-outgrowth, so far only seen *in vitro* and in microlesions (e.g., 460 μm stab wound) (Filous et al., 2010; Anderson et al., 2016; Wang H. et al., 2018; Yao, 2018; Yang et al., 2020). Nonetheless, recent studies have shown that there is a window of opportunity where the properties of the glial scar can be altered to facilitate viable regeneration of injured axons. Indeed, early interventions that decrease scar density without compromising its integrity have been shown to promote regeneration (Yang et al., 2020).

As previously mentioned, NG2+ glial cells, fibroblasts, pericytes and macrophages make up the cellular composition of the lesion core while the penumbra mostly consists of astrocytes and microglia. In order to gain insights into how to generate an environment more permissive for regeneration we need to take into account key events in glial scar formation and heterogeneity (Wanner et al., 2013; Filous and Silver, 2016; Adams and Gallo, 2018), namely astrocyte migration, proliferation, and neuro-inflammation, and the direct consequences of cell death and extracellular calcium release which occurs as a result of excitotoxicity.

### Astrocyte migration

Astrocyte migration is essential for controlling the overall astrocytic response to inflammatory factors released mostly by microglia. Astrocytic communication with the extracellular space along with inter-astrocytic communication will determine the number of astrocytes present in the glial scar, their phenotype and if they are going to be scar-forming astrocytes or reactive astrocytes (Lagos-Cabré et al., 2020). Interestingly, scar-forming astrocytes, unlike reactive and naïve astrocytes, are stationary, and they express *N*-cadherin whilst reactive astrocytes express β-catenin and metalloproteases (MMP2 and MMP13) consistent with their ability to migrate (Kanemaru et al., 2013; Verslegers et al., 2013). The inability of scar forming astrocytes to migrate may indicate that any increase in scar forming astrocytes after glial scar maturation is dependent on proliferation and differentiation of other cells types into astrocytes (Wang H. et al., 2018; Yang et al., 2020). Migration in astrocytes is controlled by gap junctions (cell-to-cell communication) and by hemichannels (cell-to-ECM communication) both of which, in astrocytes, consist of connexins (Cx). Thus, connexins are one of the most important proteins for astrocytic communication and consequently migration (Moore and O’Brien, 2015; Lagos-Cabré et al., 2020).

In astrocytes these connexions are predominantly Cx43 and Cx30. It is of interest that Cx43 is upregulated under inflammatory conditions and its activity seems to be responsible for maintaining astrocyte reactivity by promoting ATP release and calcium (Ca^2+^) cascades (Lagos-Cabré et al., 2020). On one hand, astrocyte-to-ECM communication is key in the context of the glial scar given that most roadblocks for central axonal regeneration are expressed in the ECM (Filous and Silver, 2016; Lagos-Cabré et al., 2020; Yang et al., 2020). On the other hand, migration rates are highly dependent on astrocyte-to-astrocyte communication (Moore and O’Brien, 2015; Bylicky et al., 2018), as the reactivity state of astrocytes depends not only on the astrocyte response to extracellular inflammatory cues released mainly by microglia, but also on the self-perpetuating cycle of inter-astrocytic communication. In the healthy brain, these functions of astrocytes are beneficial and are important for maintaining homeostasis (Bylicky et al., 2018). However, in the diseased or injured brain, continuous astrocytic reactivity in the context of the glial scar is highly detrimental and impedes neuro regenerative processes (Sofroniew, 2015; Anderson et al., 2016).

### Astrocyte proliferation/differentiation

Proliferation and migration rates of astrocytes in the CNS differ vastly depending on injury type and location (Haim et al., 2015; Guo et al., 2020). Astrocytes proliferate and differentiate upon injury to the CNS and can become reactive or scar forming. The balance of these cell states alter the properties of the glial scar (Wanner et al., 2013). Intriguingly other cell types can differentiate into astrocytes, or even neurons, upon injury and help redefine the cellular composition of the scar (Yang et al., 2020). For instance, ependymal cells, in stroke and SCI models of CNS injury, were found to differentiate into scar forming astrocytes (Carlén et al., 2009). In addition, NG2 + cells have been reported to differentiate into reactive astrocytes following brain injury, which is relevant because they form part of the lesion core, possibly playing a role in tissue regeneration and macrophage digestion via this differentiation process. Apart from astrocytes, it is also important to mention that different microglial phenotypes (M1, M2) may also be interchangeable at the injury site (D’Ambrosi and Apolloni, 2020; Yang et al., 2020).

The complex regulation of astrocyte proliferation and their vast heterogeneity contribute to the complexity of the glial scar. The Shh signaling pathway is one of the pathways contributing to this complexity. While suppressing astrocyte proliferation during development, Shh signaling is required for astrocytic proliferation, in a context of a stab wound injury model. This implies a stage specific role for Shh in differentiation and patterning. Moreover, different roles are attributed to Shh signaling in normal and reactive astrocytes and amongst distinct phenotypes of reactive astrocytes (Burrows et al., 1997; Wanner et al., 2013; Gallo and Deneen, 2014; Gingrich et al., 2022), therefore suggesting differing roles in reactive and scar-forming astrocytes as well.

It is relevant to point out that some pathways implicated in astrocyte proliferation are also active during differentiation. The MAPK-ERK (Mitogen-activated protein kinase-Extracellular signal-regulated kinase) pathway is considered a central signaling pathway for astrocyte generation (Cheng et al., 2013). It has also been implicated in neural stem cell (NSC) proliferation and differentiation where EGF, one of its ligands, acts through this pathway to initiate an NSC-like conversion in proliferating astrocytes (Burrows et al., 1997). This axis of astrocyte-NSC-like state is of direct relevance to axonal regeneration (Filous et al., 2010; Xu et al., 2012; Jevans et al., 2021; Zhang et al., 2021). Something that will be discussed further in the present work.

### Neuroinflammation

A neuro-inflammatory process forms an integral part of the CNS response to injury. Microglia release cytokines and inflammatory factors (e.g., Il-1α, TNF, C1q). This causes a response by reactive astrocytes to injury which can then become scar-forming astrocytes or not (Liddelow et al., 2017; Yang et al., 2020). Reactive astrocytes in turn can release cytokines and chemokines (e.g., CCL2, CCL5, CXCL8) that recruit monocytes capable of differentiating into macrophages (Mildner et al., 2009; Zhou et al., 2018). Additionally, other cytokines and inflammatory factors are overexpressed in the injured brain. These will influence the formation of the glial scar and determine its characteristics which will consequently define the extent of inhibition of axonal regeneration. One example is the cytokine Il-6 which directly influences injury outcomes by selectively converting neuronal stem/progenitor cells into astrocytes via enhanced JAK/STAT3 signaling (Yang et al., 2020). Il-6 has also been shown to increase the expression of some axonal growth promoting genes and increases mTOR expression in neurons in and around the injury site, thereby promoting neurite outgrowth (Yang et al., 2012). Thus, neuroinflammation is clearly important when considering injury to the CNS. Indeed, the characteristics of the inflammatory response, with regard as to whether it is beneficial or detrimental to repair, can be influenced by the composition of the ECM, in particular, CSPGs.

The inhibitory effects of CSPGs on axonal regeneration and plasticity are well documented. However, new roles for CSPGs concerning their involvement in neuro-inflammatory events have recently been unveiled. There is new evidence showing that they can regulate multiple aspects of the immune response in chronic inflammatory and demyelinating disorders of the CNS. In a new study by Francos-Quijorna et al. (2022), the molecular and cellular basis of these immunomodulatory effects of CSPGs were investigated. They showed that CSPGs impede the resolving phase of wound repair and that this results in the wound remaining in a chronic state. They investigated the cell types that entered the region in and around the injury site, the timing of their appearance following injury as well as their cytokine and chemokine signatures. Using SCI as an injury model, they demonstrated that CSPGs modulate both the innate and adaptive immune responses to injury in a manner that impedes wound resolution and that this occurs via multiple mechanisms. The effects were most pronounced 7 days post-injury, a stage important in the wound healing phase. In summary, they were able to show that CSPGs converted bone marrow-derived macrophages and microglia from pro-resolving M2-like phenotype to pro-inflammatory M1-like phenotype. During successful wound healing the predominant macrophage/microglia phenotype initially present is M1-like which then switches to M2-like during the wound resolving phase. This study demonstrated that CSPGs (but not their digestion products) block this switch so that the resolving phase is impaired. This process occurred via toll-like receptor 4 (TLR-4)-dependent pathway. Furthermore, M2 type macrophages were found to have much higher levels of TLR-4 on their surfaces than M1-type macrophages which is consistent with the observation that CSPGs had little impact on the phenotype of M1-like macrophages. Interestingly, the authors also demonstrated that CSPGs impact on adaptive immune response since their removal via Chondroitinase ABC (ChABC)-digestion resulted in fewer pro-inflammatory lymphocytes (Th1 and CD8+) entering the lesion site. Moreover, CSPGs were additionally shown to obstruct the clearance of pro-inflammatory cells from the lesion site and trap pro-inflammatory cytokines and chemokines, thus perpetuating the inflammatory response. All these findings significantly advance our understanding of wound repair in the CNS and give important insights into the mechanisms involved which will aid the design of novel therapies to mitigate the underlying causes of repair failure.

### Excitotoxicity

The term “excitotoxicity” refers to an overstimulation of NMDA receptor ionotropic glutamate receptors (iGluR), such as *N*-methyl-D-aspartic acid receptors (NMDAR), a-amino-3-hydroxy-5-methylisoxazole-4-propionate receptors (AMPAR) and kainate receptors (KAR) (Armada-Moreira et al., 2020) AMPAR and KAR activation contributes mainly to sodium influx, while NMDAR display a high permeability to both sodium and calcium (Mcbain and Mayer, 1994). The overload of calcium influx caused by overstimulation of NMDA receptor destabilizes the mitochondrial membrane potential (Chinopoulos and Adam-Vizi, 2006), causing higher ROS production and cell swelling, and ultimately neuronal death.

The decrease of glutamate reuptake mediated by the glial glutamate transporters is one of the proposed mechanisms to induce excitotoxicity (Gonçalves-Ribeiro et al., 2019). The EAAT1 and EAAT2 transporter are mainly present in the processes of astrocytes closely related to excitatory synaptic contact and are responsible for maintaining low levels of extracellular glutamate. If EAAT1 and EAAT2 transporter fail there will be an increase of glutamate levels in the synaptic cleft sufficient and enough to trigger the events that will lead to excitotoxicity (Gonçalves-Ribeiro et al., 2019), and this has been described in multiple neurological disorders, such as epilepsy, Parkinson’s disease (Van Laar et al., 2015), Alzheimer’s disease (Hynd et al., 2004), and Amyotrophic Lateral Sclerosis (ALS) (Van Den Bosch et al., 2006). On the other hand, astrocytes are also able to release large amounts of glutamate under specific situations (e.g., cerebral ischemia) that will also contribute to excitotoxicity (Belov Kirdajova et al., 2020).

**FIGURE 3:**
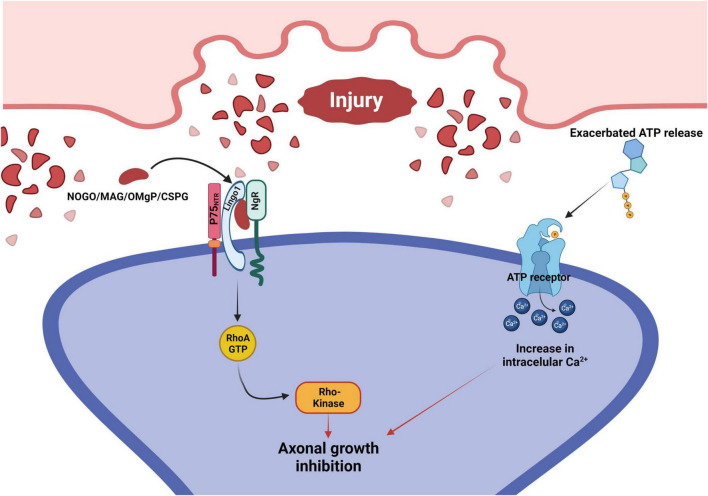
Signaling pathways that mediate axonal growth inhibition after injury to the CNS. Following an injury, there is an exacerbated release of ATP and an accumulation of growth repressive molecules on the surface of the injured neuron. Growth repressive molecules (Nogo/MAG/OMgP/CSPG) bind to the NgR, which forms a complex with both LINGO1 and P75_NTR_. This complex activates the RhoA signaling cascade, leading to the inhibition of axonal growth. At the same time, ATP binds to their neuronal membrane surface receptors, promoting intracellular Ca^2+^ increase that inhibits axonal growth. P75_NTR_, p75 neurotrophin receptor; LINGO1, leucine rich repeat and immunoglobin-like domain-containing protein 1; NgR, Nogo receptor; MAG, myelin associated glycoprotein; OMgP, oligodendrocyte myelin glycoprotein; CSPG, chondroitin sulfate proteoglycan.

Furthermore, cellular damage leads to a large release of ATP (Figure 3), which in turn activates TNFα that consequently directly affects astrocytes by inducing a slow intracellular Ca^2+^ increase, disturbing voltage dependent glial functions, and thus also increasing intracellular neuronal Ca^2+^, contributing to the overall excitotoxicity present in environments of large-scale cellular damage, such as the glial scar (Yao, 2018; Xing et al., 2019). Another effect of a sudden large release of ATP in the CNS is the stimulation of proliferation of microglia, and acting as a chemoattractant to the injury, both events increasing the already damaging inflammatory processes taking place at the injury site (Yao, 2018). Therefore, we cannot discard the concept of excitotoxicity while evaluating the possible glial scar consequences and therapeutical targets.

## Molecular pathways of regeneration

Following axotomy in the CNS, a local independent injury response takes place. There is significant upregulation of transcriptional factors (e.g., LMO4 – which interacts with the repulsive guidance molecule A receptor Neogenin; Schaffar et al., 2008) which ultimately causes inhibition of axonal regeneration. Neuroinflammatory agents are secreted in large quantities. Astrocytes become activated and growth inhibitory molecules are deposited in the ECM, in and around the lesion site. The following stand out in the molecular cascade which results in axonal growth inhibition: CSPG; myelin-associated glycoprotein (MAG); Nogo-A and oligodendrocyte myelin glycoprotein (OMgp) (Liu et al., 2006; Song et al., 2006; Schwab and Strittmatter, 2014). The three myelin-derived proteins are expressed by oligodendrocytes and increase at the lesion site arising mostly from oligodendrocyte damage and debris released upon axotomy/injury to the CNS (Liu et al., 2006; Uyeda and Muramatsu, 2020).

Intriguingly all these growth repressive molecules bind to the Nogo receptor (NgR) expressed by neurons. This receptor forms a complex with the p75 neurotrophic receptor (p75NTR) and Lingo-1 (Figure 3). The p75NTR then releases Rho guanosine nucleotide dissociation inhibitors (Rho-GDIs) from GDP bound (inactive) Ras homologous member A (RhoA) which in turn prompts the active form of GTP-bound RhoA. This cascade activates the Rho kinase (ROCK) and, as a consequence, molecules involved in cytoskeleton formation are phosphorylated resulting in overall axonal growth inhibition (Mi et al., 2004; Liu et al., 2006; Schwab and Strittmatter, 2014).

Adding to the complexity of the signaling cascades which ensue following injury, the p75NTR is not only differentially expressed by myelinating glia, but it also seems to play different roles in determining regenerative outcomes in neurons and in glia. In Schwann cells (SC) for instance, p75NTR is only overexpressed following injury. As previously discussed, the capacity for the PNS to mount a regenerative response following nerve injury is superior to that of the CNS due to the role played by SCs. Transplantation of SC and olfactory ensheathing cells (OEC) markedly enhanced axonal regeneration in SCI models (Kumar et al., 2005; Bunge and Wood, 2012). It is likely that the efficacy of SC transplantation in these models is related to the p75NTR molecular pathways and warrants their further investigation.

The p75NTR can act as a neurotrophin receptor, transducing signals which trigger either nerve regeneration (e.g., low affinity binding to BDNF, NT3/4/5, NGF) or cell death (e.g., tumor necrosis factor – TNF) (Hempstead, 2002; Uyeda and Muramatsu, 2020). However, it can also act as a co-receptor for NgR as previously discussed thus inducing growth cone collapse and growth inhibition (Wong et al., 2002; Yamashita et al., 2002; Domeniconi et al., 2005). Upon injury, neurotrophin overexpression occurs both in SC’s and other glia, yet the p75NTR is only overexpressed in SC which may thus be responsible, at least in part, for the enhanced regenerative capacity of the PNS. Indeed, it has been suggested that upon such upregulation of p75NTR in glia after injury, these receptors can function as presenting molecules for autocrine neurotrophins (Figure 1). Neurotrophins released by SC, or other glial or neuronal cells are overexpressed in and around damaged axons and may bind to p75NTR on the surface of SC. This prevents diffusion and generates a chemoattractant gradient surrounding SC’s. This is believed to attract and guide severed axons, helping them regenerate and generate synapses with their targets (Bentley and Lee, 2000; Zhang et al., 2000; Adcock et al., 2004; Song et al., 2006).

It is pertinent to remember that this is much similar to what seems to take place during CNS development, where astrocytes and radial glia function as guidance cells for neurons and a neurotrophin gradient guides axons to their target synapses (Huang and Reichardt, 2001). Some of these same neurotrophins (e.g., NGF, BDNF, NT-3/4) have been reported to promote neurite regeneration by binding to p75NTR and TrK receptors where the former seems to also increase neurotrophin affinity to the latter (Song et al., 2004, 2006; Yiu and He, 2006; Figure 1). These observations are consistent with the view that at least some of the mechanisms which regulate axon outgrowth during development are recapitulated during regeneration, further emphasizing the importance of pluripotent stem cell-like state glia (e.g., radial glia/immature astrocytes, NG2 + glia/oligodendrocyte precursors) for this endeavor.

## Barriers for central nervous system regeneration: Intrinsic and extrinsic contributing factors

### Extrinsic factors

Neuroscientists in the field have attributed the regenerative failure of CNS neurons to both intrinsic and extrinsic barriers. Two classes of molecules present in the extracellular matrix have been shown to make a major contribution to inhibition of neurite outgrowth, the CSPGs and myelin associated glycoproteins (MAGs) present on CNS myelin. Following injury, CSPGs (brevican, neurocan, phosphacan) are produced by reactive astrocytes, oligodendrocyte progenitor cells, macrophages and activated microglia (NG2 and versican) and are deposited in a glial scar (Siebert et al., 2014). Early after CNS damage, formation of the glial scar is thought to exert a beneficial function, acting to seal off the injured tissue thus protecting spared neural tissue from further damage by infiltrating inflammatory cells (Rolls et al., 2009; Sofroniew, 2015).

Reactive astrocytes play a key role in this process which is dependent on the STAT3 pathway. Supporting evidence for the involvement of the STAT3 signaling pathway in this process is that STAT3 knockout mice (KO) exhibit exacerbated damage and impaired recovery following SCI (Okada et al., 2018). Conversely, SOCS3 (which negatively impacts on this pathway) knockout (KO) mice and IL-6 (which works upstream of STAT3) expressing astrocytes exhibited improved recovery (Okada et al., 2018). However, later the mature scar is thought to act as a significant barrier to neuron regeneration (Rolls et al., 2009; Yang et al., 2020) and scar forming astrocytes are thought to be partly responsible (Okada et al., 2018). CSPGs inhibit neurite outgrowth non-specifically by sterically blocking adhesion of growth promoting molecules such as the integrins and by facilitating inhibition by trapping chemo-repulsive molecules for example sema3A. They also act specifically by binding to receptors which mediate cellular pathways involved in inhibiting axon outgrowth as discussed below.

**FIGURE 4:**
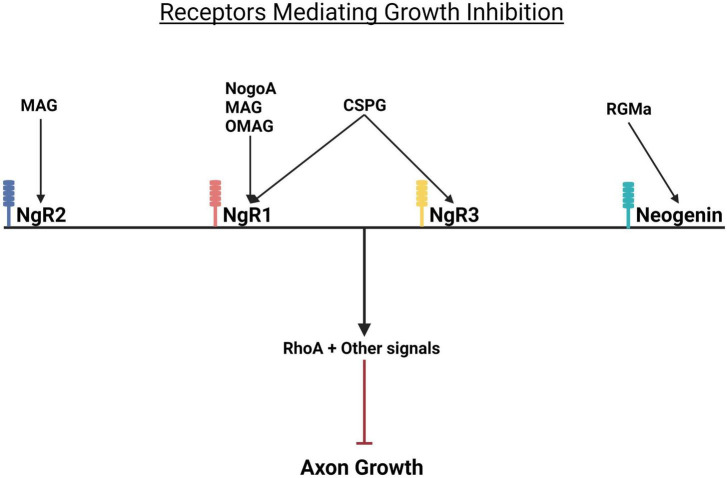
Growth repressive molecules and their receptors. Growth inhibition by CSPGs and myelin proteins is mediated by Nogo receptors. While CSPGs bind to the Nogo receptors NgR1 and NgR2, myelin inhibitors (MAG and OMgP) bind to NgR1 and NgR2. RGMa binds to its receptor neogenin. When activated, all these receptors trigger the activation of RhoA and consequent inhibition of axon growth. NgR, Nogo receptor; MAG, myelin associated glycoprotein; OMgP, oligodendrocyte myelin glycoprotein; CSPG, chondroitin sulfate proteoglycan; RGMa, repulsive guidance molecule A.

**FIGURE 5:**
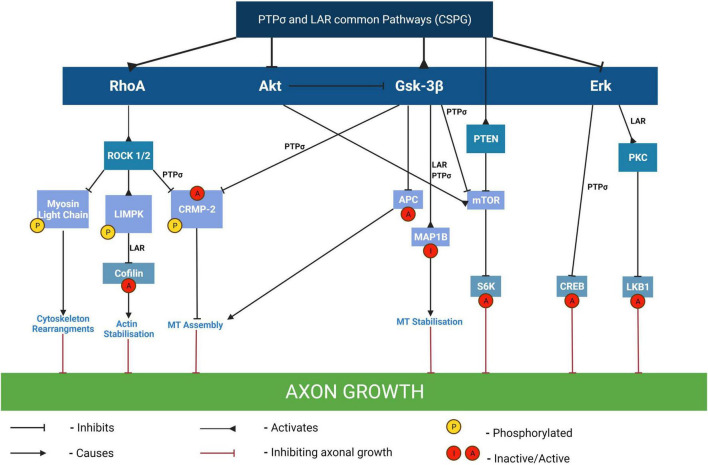
Pathways mediating the inhibitory effects of CSPGs on axon outgrowth. By binding to either PTPσ or LAR receptors, CSPGs mediate growth inhibition through modulation of several different signaling pathways including activation of RhoA, Gsk-3β and/or PTEN pathways and/or inhibition of Akt, and/or of Erk pathways. The inactivation of cofilin by phosphorylation results in the stabilization of F-actin and consequent inhibition of neurite outgrowth and is mediated by LAR. In contrast, the inactivation of CRMP-2 and APC which results in inhibition of microtubule assembly with consequent growth inhibition is mediated by PTPσ. The downregulation of Akt and Erk signaling pathways results in inactivation of their downstream targets mTOR/S6 kinase and CREB respectively, which are required for neurite outgrowth. MT, microtubules; LKB1, liver kinase B1 (which plays a role in axon growth initiation and elongation); MAP1B, MT-associated protein 1B (its inhibition results in axon growth inhibition via MT stabilization); CRMP-2, collapsin response mediator protein-2 (this is a MT interacting protein and its inactivation by phosphorylation results in growth cone collapse); APC, adenomatous polyposis coli; PKC, isoform of protein kinase C (involved in the activation of LKB1 and consequent axon growth); CREB, cyclic AMP response element binding protein (phosphorylation of CREB recruits the transcription activator CREB-binding protein to stimulate CRE-related genes involved in the generation of neurites); S6K, S6 kinase (a downstream effector of mTOR involved in protein translation and required for cell growth). Adapted from Sami et al. (2020).

Myelin associated glycoproteins present in the scar are: NogoA, MAG, and OMgp, a GPI-linked glycoprotein (Yang et al., 2020; Roy et al., 2021). The myelin proteins bind to the Nogo receptors NgR1&R2 (Figure 4). CSPGs bind to protein tyrosine phosphatase receptor sigma (PTPσ) and leukocyte common antigen-related phosphatase receptor (LAR) expressed on axons and by glia (Figure 5). More recently CSPGs have been shown to also bind to NgR1 and NgR3 (Dickendesher et al., 2012; Yang et al., 2020; Figure 4). Both the CSPGs and the myelin inhibitors activate the Rho/Rock intracellular signaling pathway which inhibits axon-outgrowth and downregulates the Akt signaling pathway which promotes axon outgrowth. There is evidence that PTPσ and LAR signal through common pathways but also employ distinct signaling pathways to exert their inhibitory effects on axon outgrowth (Figure 5). The following examples illustrate this. Activation of MAP1B which results in microtubule stabilization and consequent growth inhibition is mediated by both PTPσ and LAR whilst inactivation of LKB1, required for axon initiation, is mediated by LAR and inactivation of collapsin response mediator protein 2 (CRMP-2) and adenomatous polyposis coli (APC), which inhibit microtubule assembly is mediated by PTPσ (Figure 5). These observations are consistent with the report that PTPσ-LAR double KO elicited more axon regeneration than seen in PTPσ or LAR single knock out mice (Sami et al., 2020). Although the pathways involved in CSPG signaling have not yet been fully resolved, there is evidence that they activate the Rho/Rock, PKC, GSK-3β signaling cascades which inhibit axon outgrowth and inactivate the Akt/mTOR and Erk signaling pathways that promote cell growth and neurite outgrowth (Figure 5; Sami et al., 2020).

### Intrinsic factors

Adult mammalian CNS neurons exhibit a markedly reduced capacity to regenerate. One cause is the downregulation of the mTOR signaling pathway which plays a role in neurite outgrowth. It is downregulated during development and the levels are further reduced following CNS injury (Morgan-Warren et al., 2013). The importance of this signaling pathway and that of the JAK/STAT pathway for enhancing the intrinsic ability of neurons to regenerate is illustrated by the results of the following studies.

In 2011, a ground-breaking study from the lab of Zhigang He (Sun et al., 2011) demonstrated that co-deletion of PTEN, a negative regulator of the mTOR pathway, and SOCS3, a negative regulator of JAK/STAT signaling, in adult retinal ganglion cells (RGCs) enabled long-distance axon regeneration following a nerve crush injury. They further showed that both pathways are involved in neurite outgrowth and function synergistically to promote axon regeneration (Sun et al., 2011). In follow up experiments they demonstrated that co-deletion of PTEN and SOCS3 in the sensorimotor cortex also promoted sprouting of CST axons following SCI and that this resulted in recovery of skilled locomotor function (Jin et al., 2015). They went on to report that PTEN knockdown using a ShRNA, also proved efficacious in promoting CST-axon regeneration following SCI in rats. Axons crossed the lesion site to make functional synapses caudal to it. Thus, PTEN knockdown can also be achieved by non-genetic methods. It is of note that the axons crossing the lesion site were associated with cellular bridges likely derived from astrocytes (Zukor et al., 2013).

Moreover, researchers have demonstrated a role for PTEN in ChABC-mediated neurite outgrowth. PTEN mRNA and protein expression were significantly reduced in SH-SY5Y neurons expressing ChABC compared to GFP-transfected controls, and this was accompanied by the increase neurite length. In this study it is also shown that a similar increase in neurite length was produced by the PTEN inhibitor VO-OHpic (Rosivatz et al., 2006). This is a vanadium-based potent inhibitor of PTEN which, unlike some of the other vanadium-based inhibitors, is highly specific for PTEN (Mak et al., 2015). Moreover, it was shown that a combination of ChABC and the PTEN inhibitor did not increase neurite length further (Day et al., 2020). This implies that ChABC promotes neurite outgrowth on CSPGs via a PTEN-dependent mechanism and that CSPGs, in common with myelin inhibitors (Perdigoto et al., 2011), block neurite outgrowth, via a pathway involving PTEN.

The importance of the JAK/STAT pathway for neurite outgrowth of the corticospinal tract (CST) is supported by the observation that overexpression of STAT3, achieved by fusion with the viral activation domain VP16, enhanced neurite outgrowth of cultured primary cortical neurons (Mehta et al., 2016) and consistent with the fact that STAT3 is elevated in injured neurons of the PNS which regenerate but not the CNS which do not regenerate (Liu et al., 2015). Also consistent with this view, STAT3 deletion in DRGs of STAT3 floxed mice impairs regeneration of the peripheral nerve branch following a nerve cut and its overexpression in the CNS branch results in significantly enhanced regeneration (Bareyre et al., 2011). Interestingly, the authors identify a role for STAT3 in initiation but not elongation of axon-outgrowth, suggesting that it is involved in kick starting regeneration (Bareyre et al., 2011).

Furthermore, a study investigating genes involved in regeneration following optic nerve crush identified IL-22 as a key player in the process. They used shRNA knockdown and CRISPR gene editing to show that knockdown or inactivation of IL-22 resulted in the upregulation of two pro-regenerative signaling pathways; STAT3 and DLK, and this enhanced regeneration. The results support their hypothesis that reduced levels of functional IL-22 results in dis-inhibition of inflammation after nerve crush. This leads to increased levels of growth promoting transcription factors which in turn stimulate both STAT3 and DLK signaling pathways. This culminates in expression of regeneration associated genes (RAGs) and associated enhanced regeneration (Lindborg et al., 2021). SOCS3 is a negative regulator of JAK/STAT signaling, as discussed above, and its expression is induced by activation of the JAK/STAT pathway forming a feedback loop of inhibition. Using SCI as an injury model Liu et al. (2015) showed that the JAK/STAT pathway is activated early after injury and that, as expected, this is followed by a rise in the levels of SOCS3. SOCS3 binds to JAK attenuating their response to cytokines and growth factors, thus limiting the regenerative response resulting from the JAK/STAT pathway activation. Interestingly, SOCS3 knockdown elicits an anti-apoptotic effect following injury implying an additional role for STAT3 in neuronal cell survival as well as regeneration (Liu et al., 2015).

This association between inflammation and nerve regeneration is also documented by Yin et al. (2003) and Kurimoto et al. (2010). Using an optic nerve crush injury, they showed zymosan-induced inflammation in the optic nerve resulted in robust regeneration by enhancing the intrinsic ability of nerve to regenerate. This was attributed to factors secreted by activated macrophages, oncomodulin, in particular (Kurimoto et al., 2010). However, the role of activated macrophages was later challenged by Hauk et al. (2008) who suggested a possible role for activated astrocytes or monocytes. Whatever the mechanism responsible, zymosan is a potent enhancer of the intrinsic ability of RGCs to regenerate.

Most studies have focused on knocking down molecules which inhibit intrinsic signaling pathways that promote axon-outgrowth, namely PTEN and SOCS3. However, others have taken the approach of boosting the activity of these pathways. Another way to activate mTOR is by activating the GTPase Rheb which acts upstream of mTOR. When constitutively active (ca–) Rheb was delivered to a ChABC-treated injury site of rats with SCI, together with a peripheral nerve graft (PNG), axons, mostly of propriospinal origin, grew out of the graft into the gray matter where there was evidence of synapse formation (Wu et al., 2015). In a later study, the same group showed that delivery of ca-Rheb + PNG to a C2 hemisection injury resulted in more axons growing out of the graft into the spinal gray matter and their data is consistent with those axons making functional connections as evidenced by cFos expression following graft stimulation (Wu et al., 2017).

In an innovative study by Nieuwenhuis et al. (2020), the same pathway is targeted with the aim of promoting axon regeneration. Their hypothesis is that one cause for the low regenerative capacity of adult CNS neurons, particularly cortical neurons, is due to low cellular levels of PIP3. This is consistent with the observation that high levels of PIP3 are present in the axons of regenerating immature axons but that the levels drop substantially in mature neurons which do not regenerate (Nieuwenhuis et al., 2020).

They identify two subunits of the enzyme PIP3 kinase as important for axon growth and regeneration of cortical neurons. They are P110α and P110δ. P110α is required for growth and acts via mTOR in the cell soma whilst P110δ is needed for a regenerative response and acts in both the soma and axonal compartments. Forced expression of this isotype of PIP3 kinase in cortical neurons *in vitro* or in an optic nerve crush injury *in vivo* resulted in significant nerve regeneration (Nieuwenhuis et al., 2020). Expression of hyper activated P110α (H104R) or overexpression of p110δ resulted in enhanced axonal PIP3 levels. It is thought that the regenerative effects are at least in part due to PIP3 inactivating the GTPase ARF6 which promotes retrograde trafficking in the axon thereby inhibiting anterograde transport of cargo required for regeneration, such as integrins. Interestingly, when the effects of PTEN knockdown (via shRNA) were compared to the effects of P110δ overexpression, the latter was more effective at promoting axon-outgrowth. The authors suggest that this may be because the levels of PIP3 present are so low in adult neurons that PTEN knockdown has little effect. This would be consistent with the observation that PTEN knockdown is more effective at promoting neurite outgrowth in immature neurons. Thus, P110δ provides a novel target for therapeutic intervention and supports the accumulating evidence that targeting certain therapeutic molecules to the axonal compartment may provide a more effective way of promoting axon-outgrowth than targeting the cell as a whole (Nieuwenhuis et al., 2020).

**FIGURE 6:**
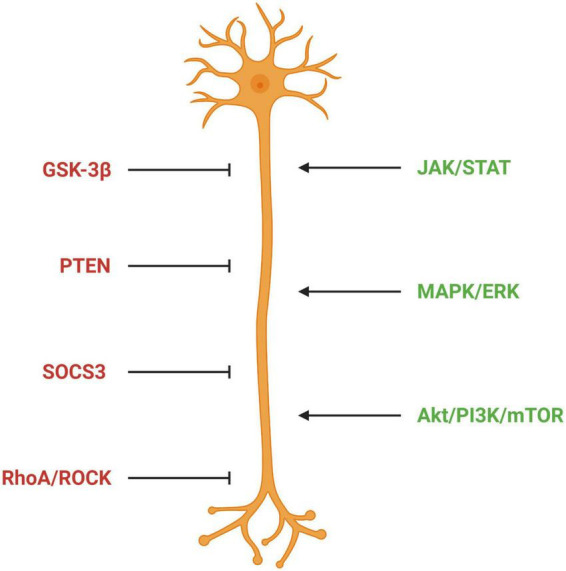
Signaling pathways regulating axon outgrowth. Factors in red represent pathways inhibitory for axon outgrowth; factors in green represent pathways permissive for axon outgrowth. GSK-3β, glycogen synthase kinase 3 beta; PTEN, phosphatase and tensin homolog; SOCS3, suppressor of cytokine signaling 3; ROCK, Rho-associated protein kinase; JAK/STAT, Janus kinase/Signal transducer and activator of transcription; MAPK/ERK, mitogen-activated protein kinase/extracellular signal-regulated kinase 1/2; Akt/PI2K/mTOR, protein kinase B/phosphatidylinositol-3-kinase/mammalian target of rapamycin.

In summary, these studies identify the Akt/mTOR and JAK/STAT signaling pathways as key players in regulating the potential of CNS nerve cells to regenerate. They also show that activating these pathways by knockdown of molecules that inhibit them or by activation of components essential to their function can promote regeneration (Figure 6). This makes them attractive therapeutic targets. Preclinical studies using some of these approaches are discussed in the quest for axon regeneration section (see section “The quest for axonal regeneration”).

### Synapse-related molecules

Pursuing a novel avenue of research Hilton et al. (2022) identify synaptic vesicle priming proteins of the presynaptic active zone as key inhibitors of axon regeneration. They used whole transcriptome analysis to identify genes associated with growth competence. They analyzed genes upregulated or down regulated at mouse E12.5 and E17.5 (Hilton et al., 2022), a time when embryonic neurons stop elongation and start to form synapses. They also analyzed DRGs following a conditioning lesion and cultured DRGs which acquire the ability to grow axons with increasing time in culture. They show that whilst there was no correlation seen in the upregulated genes, a set of downregulated genes was consistent between the groups. Gene ontology revealed that these were related to synaptic function. Gene knock out studies revealed that MUNC 13 the effector through which the Rab3 interacting molecule RIM regulates vesicle docking/priming and fusion was the most potent inhibitor of axon outgrowth and that MUNC13 KO mice exhibited axon regeneration following SCI. Moreover, they demonstrated that decreasing excitability of the neurons with the DREADD receptor hm4Di and clozapine also enhanced axon regeneration and showed that Baclofen, a GABAB receptor agonist that decreases synaptic transmission, stimulated sensory axon regeneration following SCI. Thus, MUNC13 is a developmental switch, which inhibits growth and regeneration in maturing neurons. Moreover, it has also been shown that the voltage-gated calcium channels (VGCCs) subunit Alpha2delta2 (Cacna2d2) restrains axon growth at late stages of embryonic development and impairs axon regeneration in the adult CNS (Tedeschi et al., 2016). There is also evidence that an intact axonal branch can suppress regeneration (Lorenzana et al., 2015), which is consistent with the hypothesis that synapse-related molecules impact on regeneration.

## Microtubule dynamics: The overlooked underdog underlying axonal regeneration

The cytoskeleton of eukaryotic cells is composed of three structurally, morphological and functionally distinct types of polymeric cytoplasmic proteins, namely microtubules, microfilaments, and intermediate filaments (Mohan and John, 2015). The microtubule cytoskeleton constitutes a central structural element that controls, among other functions, neuronal morphology and supports the establishment, maintenance, and plasticity of axonal connections with cell targets. Microtubules are critically important for early neuronal developmental stages, such as in cell migration, neurite outgrowth and the cue-dependent navigation of the elongatingaxon (Kapitein and Hoogenraad, 2015; Matamoros and Baas, 2017). This is possible because at the distal tip of the axon there is a specialized compartment that contains a cytoskeletal machinery that dynamically changes in response to extracellular guidance or positional cues. This motile structure has a central domain rich in microtubules which is surrounded by actin filaments in the peripheral domain (Gordon-Weeks, 2004; Lowery and Van Vactor, 2009). Between these domains, actomyosin forms the transition zone, which may restrain dynamic microtubules from protruding into the peripheral domain (Dupraz et al., 2019).

Microtubules are polymers of tubulin heterodimers of α-tubulin and β-tubulin subunits uniformly oriented. This structural polarity confers a different dynamic to microtubule ends. In axons, microtubule fast-growing ends, the plus ends, are nearly directed away from the soma (Matamoros and Baas, 2017). During neuronal development, microtubule plus end cycle through periods of slow and continuous growth and periods of rapid shrinkage in a phenomenon called dynamic instability (Cassimeris et al., 1987). The change from microtubule growth to rapid shrinkage is named catastrophe, while the opposite is designated rescue (Gordon-Weeks, 2004). Apart from being labile, microtubules also possess stable regions and cold-stable regions. While stable microtubules are still dynamic, although more slowly compared to the labile microtubules, the cold-stable category is so stable that seems to not undergo dynamics (Matamoros and Baas, 2017). The more stable, the less the rate at which a microtubule undergoes subunit exchange with the soluble tubulin pool (Baas et al., 2016).

The dynamic properties of microtubules are mainly due to post-translational modifications of tubulin, which play an important role in regulating microtubule properties, such as stability and structure, as well as microtubule-based functions and interaction with other cellular components (Hammond et al., 2008). Among post-translational modifications are detyrosination/tyrosination and acetylation, which constitute important regulators of the neuronal microtubule cytoskeleton (Fukushima et al., 2009; Moutin et al., 2021). Acetylated tubulin is enriched in stable, long-lived microtubules along the axon shaft, correlating with microtubule stability, whereas tyrosinated tubulin is enriched in the growth cone area, correlating with highly dynamic and recently synthesized microtubules in that region. Therefore, while labile microtubule regions are deficient in acetylated and detyrosinated tubulin, the stable regions are rich in acetylated and detyrosinated tubulin (Fukushima et al., 2009; Moutin et al., 2021). Cold-stable tubulin can be attributed to polyamination (Song et al., 2013). While most modifications make proteins more acidic or are neutral, polyamination, catalyzed by transglutaminases, is known to make proteins more basic, causing proteins to become stable and insoluble, a process that is known to increase as neurons mature (Song et al., 2013). Although both developing and adult neurons contain stable and dynamic microtubule regions, there is a higher percentage of stable regions in adult than in developing neurons, which are correlated with an increase of acetylated microtubules (Ferreira and Cáceres, 1989; Lim et al., 1989).

When speaking about the neuronal cytoskeleton dynamics, microfilaments, or actin filaments, are a major protein structure to consider. Actin is a conserved protein expressed ubiquitously in cells. It may be present as a monomer or a filament (Bradke and Dotti, 1999; Blanquie and Bradke, 2018). ATP-bound G-actin (monomer) may polymerize into F-actin (filament). F-actin is an intrinsically dynamic and polarized structure with a plus- and a minus-end actively undergoing dynamic processes (Lin and Forscher, 1995). In cells in general, actin filaments are considered to be responsible for cell polarity, tissue organization, motility and cell division (Gordon-Weeks, 2004; Blanquie and Bradke, 2018). However, in neurons, microfilaments form structures of lamellipodia and filopodia which by interacting with microtubules are essential for growth cone motility and guidance (Gomez and Letourneau, 2014).

While axons in the CNS do not regrow after an injury, lesioned axons in the PNS can regenerate, as aforementioned. In response to the extracellular environment, it is necessary that injured neurons transform their damaged axonal end into a new growth cone-like structure in order to initiate regeneration. However, after lesion, CNS axons often fail to reform a growth cone and instead form a retraction bulb, which contains a disorganized microtubule network which inhibits axon growth (Ertürk et al., 2007). In fact, cerebellar granule neurons (CGNs) plated on CSPGs have their growth cones reduced in size, with fewer lamellipodia and less dynamic, when compared to a laminin substract (Stern et al., 2021). A physiological mediator of these actions of CSPGs is Ras homolog family member A (RhoA), which is a small GTPase protein in the Rho family of GTPases. RhoA inhibits growth by restraining growth cone dynamics (Dupraz et al., 2019; Stern et al., 2021). Depending on the GTPase being expressed, actin filaments and structures are going to be different (e.g., Rar1 results in an actin arrangement called lamellipodia) (Pinto-Costa et al., 2020). RhoA on the other hand results in actin structures named stress fibers, which are partly responsible for retraction bulb formation (Dupraz et al., 2019; Stern et al., 2021). CSPGs and myelin-associated inhibitors signal through their receptors to converge on RhoA activation, preventing microtubule protrusion to the growth cone leading edge and, thereby, axon regrowth in the lesioned CNS (Fujita and Yamashita, 2014; Stern et al., 2021). RhoA restrains axon growth by activating actin arc formation through the actin motor myosin II, by phosphorylation of the myosin light chain (MLC), to induce F-actin compaction which prevents microtubules from protruding toward the growth cone leading edge (Dupraz et al., 2019; Stern et al., 2021). In fact, RhoA ablation in neurons allows axon regeneration through a defined cellular cascade that ultimately enables microtubule protrusion in the axon tip, propelling it forward (Dupraz et al., 2019; Stern et al., 2021). Therefore, modulating cytoskeleton dynamics is essential not only to regulate axon growth and guidance during development but also to growth cone formation and dynamics following injury (Blanquie and Bradke, 2018).

Post-translational tubulin modifications are relevant for axonal regeneration as it regulates cytoskeleton dynamics. A variety of post-translational modifications were described to occur following SCI, such as tyrosination, acetylation, and phosphorylation (Zhu et al., 2022). Axon injury induces a gradient of tubulin deacetylation, reducing stable microtubules in proximity of the injury site, an effect that is necessary for growth cone dynamics and axon regeneration, and specific to peripheral neurons, failing to occur in central neurons. α-Tubulin deacetylation in PNS axons is initiated by calcium influx at the site of injury, and requires protein kinase C (PKC)-mediated activation of the histone deacetylase 5 (HDAC5) (Cho and Cavalli, 2012). Such a reduction in microtubule acetylation does not occur in the CNS, suggesting that tubulin modifications that accompany microtubule stability negatively impact the capacity of the axon to regenerate (Zhu et al., 2022). In fact, HDAC5 knockdown and inhibition restricts growth cone dynamics and regeneration of dorsal root ganglion (DRG) neurons both *in vitro* and *in vivo* and in CGNs, whereas HDAC5 overexpression had the opposite effect (Rivieccio et al., 2009; Gaub et al., 2010; Cho and Cavalli, 2012). The converse reaction, acetylation, is mainly catalyzed by tubulin acetyltransferase αTAT1. Both CSPGs- and MAG induce a reduction in αTAT1 mostly in the distal and middle regions of neurites and reconstitution of αTAT1 can restore neurite growth (Wong et al., 2018).

Accordingly, a popular preclinical strategy for altering the microtubule system toward axonal regeneration has been to stabilize microtubules by increasing microtubule acetylation and other tubulin modifications (Zhu et al., 2022). Microtubule-stabilizing drugs, such as taxol and epothilones have shown to have a positive impact in the regenerative capacity of injured adult CNS axons, by reducing the inhibitory fibrotic lesion scar and enhancing the capacity of axons to grow (Ertürk et al., 2007; Ruschel et al., 2015; Ruschel and Bradke, 2018; Sandner et al., 2018). Microtubule stabilization facilitates axonal growth and desensitizes growth cones toward inhibitory molecules *in vitro* (Sengottuvel and Fischer, 2011). Nevertheless, there are concerns that the negative consequences of abnormal microtubule stabilization may outweigh the positive effects, especially if the drugs are taken systematically (Baas and Ahmad, 2013; Matamoros and Baas, 2017).

A decrease in the proportion of labile microtubules with aging and the importance of the different microtubule domains, labile and stable, especially the dynamic properties of microtubules at the distal tip of the developing axon, have resulted in the proposal of an alternative approach for augmenting regeneration of injured CNS axons. Instead of stabilizing existing microtubules, the idea is to increase the amount of labile microtubule mass in the adult axon, especially in its distal area near the growing tip. A strategy to increase more labile microtubule mass to the regenerating axon would be to deplete or inhibit proteins that suppress the expansion of labile domains (Matamoros and Baas, 2017). One such protein is fidgetin, a microtubule-severing protein that targets labile domains and is specific for non-acetylated tubulins (Leo et al., 2015; Austin et al., 2017). Knockdown of fidgetin is sufficient to increase the content of microtubules in the axon, with a greater fraction of which being labile and less acetylated, thus increasing axonal growth in cultured adult rat DRG neurons, both in favorable and non-favorable containing aggrecan substracts (Austin et al., 2017; Matamoros et al., 2019). In addition, in *in vivo* experiments of adult female rats, contrary to control DRG neurons, fidgetin knockdown enhanced regeneration of axons across the dorsal root entry zone into the spinal cord after injury (Matamoros et al., 2019). By increasing labile microtubule mass, microtubules become more similar to those in juvenile axons during development, which can be an advantage for axon regeneration.

On the other hand, microtubule-severing proteins, such as katanin and spastin, which prefer stable domains, namely regions of microtubules that are rich in tubulins that have been post-translationally acetylated or polyglutamylated (Lacroix et al., 2010; Sudo and Baas, 2011), when suppressed, result in deleterious effects on axonal growth and branching (Ahmad et al., 2000; Karabay et al., 2004). These effects are due to the fact that they are responsible for generating new microtubules via the severing of existing ones.

Among proteins that contribute to microtubule stability in neurons are the microtubule-associated proteins (MAPs) such as tau and MAP1B. These MAPs are more enriched on the labile domains extending into the distal region of growing axons (Tucker et al., 1988; Schoenfeld et al., 1989), playing an important role in axon outgrowth and guidance (Meixner et al., 2000; González-Billault et al., 2002). Moreover, fibroblast growth factor 13 (FGF13), is an endogenous microtubule stabilizer that has the capacity to promote axonal regrowth, remyelination, and functional reinnervation after sciatic nerve transection injury by promoting tubulin polymerization (Li R. et al., 2021). Also, collapsin response mediator protein 2 (CRMP-2) signaling is described to stabilize cytoskeletal polymerization. When phosphorylated by Cdk5 and GSK3β, CRMP-2 loses its affinity for cytoskeleton proteins, leading to the inhibition of axonal growth and promoting sprouting of motor and sensory axons (Fukata et al., 2002; Jin et al., 2016).

Apart from what we have described, there are much more proteins with the role of modulating cytoskeleton dynamics and therefore constitute a promising target to promote axon regeneration following an injury. However, much more is to be discovered as this is a field that remains poorly understood and often neglected when considering regeneration of CNS axons in detriment for more cell/pathway directed therapeutic approaches. Rather, we would say that the overlap of some of the molecular pathways involved in regeneration discussed below with the impacts that CNS injury has on microtubule dynamics, described above, are a testament to the necessity to complement neuronal regenerative studies with microtubule dynamics analysis.

## The quest for axonal regeneration

Given both the number of processes occurring simultaneously and the heterogeneity of these events depending on injury severity and type (Adams and Gallo, 2018; Wang H. et al., 2018), it is not unexpected to find that there is an equivalent heterogeneity in the types of therapeutic approaches taken by researchers to overcome this regenerative failure. Therefore, in the following section we will be discussing some of the most promising paths of research used to promote CNS axonal regeneration while objectively analyzing the possible shortcomings and molecular basis for the interventions tested. We will focus on results generated using preclinical models of CNS injury.

### Identification of the steps required to achieve successful regeneration

In a landmark study and as proof of principal, Anderson et al. (2018) demonstrated that robust regeneration of propriospinal neurons following complete SCI in mice and rats required three interventions. (1) Activation of pathways involved in neuronal intrinsic ability to regenerate, (2) induction of a growth supporting substrate, and (3) chemo-attraction to guide regenerating axons to their targets. In this study the regenerative capacity of the neurons was activated prior to SCI by expression of osteopontin, insulin-like growth factor 1 (IGF1) and ciliary neurotropic factor (CTNF). The authors had previously established that a combination of osteopontin and growth factors enabled regeneration of RGCs following nerve crush by activating the mTOR pathway of neurite outgrowth (Duan et al., 2016). Adding fibroblast growth factor 2 (FGF2) and epidermal growth factor (EGF) to this cocktail stimulated the production of fibronectin, laminin and collagen, substrates that favor regeneration. Glial derived neurotrophic factor (GDNF) was used as the chemotactic agent.

They report that propriospinal axons crossed the glial scar, lesion site and made functional connections with spared matter caudal to the lesion site. Interestingly, regeneration was further enhanced when a second dose of GDNF was delivered 1-week post-injury one segment caudal to the first site of delivery suggesting that spatial and temporal factors are important. Following the first dose of GDNF delivered to the first segment caudal to the injury site, axons grew in and around the hydrogel used for GDNF-delivery but not beyond. This is interesting because it is similar to the response of regenerating PNS neurons where constitutive expression of GDNF resulted in nerve entrapment whilst timed expression (1 month) resulted in successful regeneration (Eggers et al., 2019). The Anderson study (Anderson et al., 2018) also highlights the fact that interventions to promote regeneration will likely need to be tailored to the neuron subpopulation in question because they showed that whilst osteopontin + growth factors resulted in upregulation the mTOR pathway in propriospinal neurons, in contrast to what was observed in RGCs, PTEN knockdown was ineffective. Moreover, it is also of note that although robust nerve regeneration resulted in a return of nerve conduction to 25% of preinjury levels there was no locomotor recovery. This indicates a need for some form of rehabilitation such as treadmill training and/or electrical stimulation to reinforce locomotor pathways.

### Interventions that boost intrinsic growth promoting signaling pathways

In another approach, transcription factors required for axon growth during development and down regulated in adult neurons or differentially expressed between the PNS and CNS have been used to promote regeneration following injury. Blackmore et al. (2012) delivered Kruppel-like factor 7 (KLF7) to the motor cortex of mice with dorsal hemisection injuries via adeno-associated viruses (AAVs) (Blackmore et al., 2012). It was found that this enhanced the growth of CST axons beyond the lesion when compared with controls. In another study (Wang Z. et al., 2018) delivered another member of this family, KLF6, to the motor cortex of animals with unilateral pyramidotomy, similarly using AAV vectors (Wang Z. et al., 2018). Transduction of neurons on the intact side resulted in enhanced CST sprouting and the axons crossed the spinal cord to the injured side. Transduction on the injured side resulted in CST growth beyond the injury site. The authors then went on to analyze the promoters of genes activated by KLF6 and identified STAT3. Again, this is consistent with this family of transcription factors playing an important role in the regeneration of CST axons and consist with the JAK/STAT signaling pathway playing a critical role. The results from this section are summarized in Tables 1A,B.

**TABLE 1A:** Counteracting the effects of scar-derived inhibitors.

Targeted inhibitor	Method	Outcome	Model	References
CSPGs GAG chains	ChABC	Anatomical and functional recovery	Rodent, Cat, Dog, and Primate SCI	Bartus et al., 2014; Mondello et al., 2015; Hu et al., 2018; Rosenzweig et al., 2019
CSPG GAG chains	Axon-targeted ChABC	Enhanced Neurite length and sprouting ↓PTEN ↓Axonal RhoA	Neuronal cell line plated on CSPGs	Day et al., 2020
CSPG GAG chains	Remove sulfate moieties with chondro-4 sulfatase	Enhanced neurite out growth	Mouse cerebellar and cortical neurons *in vitro*	Sami et al., 2020
CSPG core proteins	ADAMTS-4 + hind limb rehab	Anatomical and functional improvements	Rodent SCI	Griffin et al., 2020
CSPGs	Receptor blockade: PTPσ mimetic peptide LAR mimetic	Improved functional recovery Improved growth of 5 HT fibers	Rodent SCI	Sami et al., 2020
GSK-3β	Lithium/Ibuprofen	Improved axon regeneration and functional recovery in rodents but not patients.	Rodent SCI	Sami et al., 2020
ATP	Epac2	Increased cAMP reducing astrocyte/microglial activation Astrocytes elongated processes guiding axons	Rodent SCI	Guijarro-Belmar et al., 2019
Rho A (mediates both CSPG and myelin signals)	Rho A inhibition with cethrin/Ibuprofen	Promotes axon regeneration and functional recovery	Rodent optic nerve crush and SCI.	Sami et al., 2020
Rock 2	Y27632/NSAIDS	Regeneration, functional recovery improved outcomes	Optic nerve crush, SCI, AD, PD, HD, ALS, SMA	Fujita and Yamashita, 2014
RGMa	Anti RGMa antibodies	Regeneration and functional recovery.	SCI	Fujita and Yamashita, 2014
NogoA	AntiNogo antibodies IN1/NogoR peptide antagonist NEP-1	Axon regrowth and functional recovery	SCI	Fujita and Yamashita, 2014

AD, Alzheimer’s disease; PD, Parkinson’s disease; HD, Huntington’s disease; ALS, amyotrophic lateral sclerosis; SMA, spinal muscular atrophy; NSAIDS, non-steroidal anti-inflammatory drugs; RGMa, repulsive guidance molecule A.

**TABLE 1B:** Interventions targeting neuronal pathways involved in regeneration.

Target	Method	Outcome	Model	References
PTEN + SOCS3	Deletion	Enhanced mTOR and JAK/STAT signaling/Long distance regeneration	Optic nerve crush	Sun et al., 2011
PTEN + SOCS3	Deletion	CST sprouting/functional recovery	SCI	Jin et al., 2015
STAT3	Overexpression	Enhanced regeneration	Cortical neurons	Mehta et al., 2016
STAT3	Overexpression	Enhanced regeneration	DRGs	Bareyre et al., 2011
IL-22	Knockdown	Upregulation of STAT3 and DLK signaling Enhanced regeneration	Optic nerve crush	Lindborg et al., 2021
PTEN	Knockdown ShRNA	CST regeneration	Rat SCI	Zukor et al., 2013
PTEN	rAAV retro ShRNA	Regeneration of multiple pathways affected by SCI	Mouse SCI	Metcalfe et al., 2022
PTEN	Inactivating peptides	Enhanced CST and raphespinal regeneration	SCI	Bhowmick and Abdul-Muneer, 2021
mTOR	caRheb + PNG	Enhanced regeneration/ propriospinal axons	SCI	Wu et al., 2015
PIP_3_Kinase	Axonal expression of P110δsubunit	Enhanced regeneration of cortical spinal neurons *in vitro* + retinal ganglion cell neurons *in vivo*	Optic nerve crush	Nieuwenhuis et al., 2020.
KLF7 (regenerative associated transcription factor)	AAV KLF7 to motor cortex	Enhanced regeneration of CST	SCI	Blackmore et al., 2012
JAK/STAT signaling pathway	Overexpresstion of mi-RNA 125b	Enhanced JAK/STAT signaling and regeneration	SCI	Li Z. et al., 2021
PTEN	Knockdown with mi-19a	Enhanced mTOR signaling and regeneration	Optic nerve crush	Li Z. et al., 2021
Sema 3A	Overexpression of miRNA-30b	Sema3A growth cone collapsing activity blocked and enhanced regeneration	Optic nerve crush	Li Z. et al., 2021
KLF-4 (potent inhibitor of optic nerve growth)	Knockdown with mi-RNA-135s	Enhanced optic nerve regeneration	Optic nerve crush	Li Z. et al., 2021
mTOR signaling, fibronectin and laminin production, GDNF-induced chemotaxis	Combination therapy Enhanced mTOR signaling + stimulation of production of regenerative substrates + chemotaxis	Robust regeneration of propriospinal neurons	SCI	Anderson et al., 2018

### Therapies that counteract the inhibitory components of the glial scar

Administration of antibodies to Nogo A following SCI have produced anatomical and functional recovery in both rats and macaques and are about to re-enter clinical trials (Mohammed et al., 2020). It is of note that the timing of this intervention markedly influences outcomes. Acute delivery and delivery for up to one-week post-injury were found to be efficacious but after this time period the efficacy diminished (Gonzenbach et al., 2012).

The sugar chains present on the CSPGs are thought to be responsible for much of their inhibitory effect on axon outgrowth (Siebert et al., 2014) and targeting them for removal promotes neurite outgrowth (Bradbury et al., 2002; Bartus et al., 2014).

The GAG chains can be removed by digestion with the enzyme ChABC isolated from the bacterium *Proteus Vulgaris* and injections of the enzyme into spinal cord lesions results in anatomical and functional recovery in rats (Bradbury et al., 2002). Since this seminal study these results have been replicated by many laboratories and in several species including mice (Lee et al., 2012), cats (Mondello et al., 2015), and non-human primates (Rosenzweig et al., 2019). Moreover, a clinical trial involving dogs with SCI produced encouraging results (Hu et al., 2018). More recently, ChABC has been delivered by gene therapy. This method overcomes the inherent problem of the enzyme’s instability (Mondello et al., 2015) as transduced cells continuously produce active enzyme. Additionally, delivery of the enzyme to large areas of the cord can be achieved (Bartus et al., 2014) which is likely to be necessary for efficacy in humans where the dimensions of the cord are several orders of magnitude larger than those of rodents. Delivery of the enzyme via gene therapy also enables long-term treatment. Treatment of 8 weeks duration was required to achieve optimal levels of recovery in rats with SCI (Burnside et al., 2018). Moreover, ChABC has been shown to exhibit pleotropic effects on the damaged CNS. It is neuroprotective (Carter et al., 2008) and promotes plasticity allowing spared neurons to take over the function of damaged neurons (Zhao et al., 2011). This is of particular relevance for SCI since most injuries are incomplete. ChABC also has immunomodulatory properties which markedly reduce the amount of secondary damage which normally ensues following SCI (Bartus et al., 2014; Didangelos et al., 2014). It also promotes wound resolution by digesting CSPGs. This results in a bias of microglial phenotype from M1-like pro-inflammatory type to favor the M2-like phenotype which promotes wound healing (Francos-Quijorna et al., 2022). Furthermore, axon outgrowth is further enhanced by targeting ChABC to the axonal compartment (Day et al., 2020). In this study, using SH-SY5Y neurons which, in common with CST neurons have CSPGs located on their surface (Lander et al., 1998) we showed that removal of these inhibitory molecules by ChABC-digestion resulted in up-regulation of β1- integrins on the cell surface. Since CSPGs are known to down-regulate cell surface integrin expression (Orlando et al., 2012) and to inactivate integrins (Tan et al., 2011) which are required for regeneration (Andrews et al., 2009), this provides a likely mechanism for the ChABC-mediated effect on neurite outgrowth. It is interesting to note the CSPG effect on integrins, since reactive astrocytes (major component of the glial scar) increase drastically the expression of integrins upon injury to the CNS (Lagos-Cabré et al., 2020). It was also noted that RhoA staining is reduced in neurons expressing ChABC (non-targeted) and this becomes detectable in the axonal compartment when compartment when neurons are transfected with the construct where ChABC is targeted to the axon. CSPGs promote the translation of RhoA in axons (Walker et al., 2012) and since RhoA is a potent inhibitor of axon regeneration (Kopp et al., 2012) this may be another key mechanism by which CSPGs block neurite outgrowth. It is of interest, also that targeting a soluble form of adenyl cyclase to the axonal compartment of rat dorsal root ganglion cells (DRGs) promotes neurite outgrowth on CSPGs (Walker et al., 2012). This raises the possibility, that targeting certain growth-promoting molecules to the axon, rather than the cell as a whole, may be a more effective approach for promoting regeneration.

Removal of CSPGs can also be achieved using a different class of enzyme. ADAMTS4, a member of a class of enzymes called a disintegrin and metalloproteinases with thrombospondin motifs (ADAMTS). These target the protein core components of the CSPGs. ADAMTS4 specifically targets CSPGs (Kelwick et al., 2015). When recombinant ADAMTS4 was administered, via intrathecal injection, to rats with SCI (contusion injuries), improvements in locomotor function were reported (Tauchi et al., 2012). In another study ADAMTS4 was delivered to rats with SCI (T10 contusion) using a gene therapy approach and gene expression was restricted to astrocytes using a truncated GFAP promoter. The authors reported that removal of CSPGs resulted in a large decrease in lesion size which was accompanied by enhanced sprouting of the CST tract (Griffin et al., 2020). These results are encouraging and confirm that ADAMTS4 can be used as an alternative to ChABC for CSPG removal. The outcomes in this study are similar to those reported following removal of CSPG GAG chains using ChABC (Bartus et al., 2014) thus upholding the view that removal of CSPGs at the lesion site limits secondary damage and promotes regeneration. However, since the efficacy of ChABC in many different CNS injury models is well documented, and under some circumstances ADAMTS4 has be shown to be associated with neuro-inflammation, it currently makes ChABC the enzyme of choice for CSPG removal.

An alternative to CSPG removal is to target the downstream signaling pathways through which they act to block neurite outgrowth. The activation of RhoA has been targeted using cethrin a potent RhoA inhibitor. Lithium has been used to block GSK-3β. The dose of lithium is an important factor determining outcomes because low doses promote axon outgrowth whilst higher doses are inhibitory. The results obtained in preclinical studies were encouraging but the outcomes in clinical trials to date have been disappointing. Other RhoA and GSK-3β inhibitors such as Ibuprofen are being assessed (Sami et al., 2020).

#### Targeting the intracellular signaling pathways with ncRNAs and RNA binding proteins

There is accumulating evidence that non-coding RNA’s (ncRNAs) are dysregulated following CNS injury (reviewed at length in Li P. et al., 2021). They fall into three classes lncRNAs which are >200 nt in length, miRNAs which are 20–25 nt in length and circ RNA which form looped structures and which unlike the former two do not have cap structures at the 5’end or 3’end structures. miRNAs regulate gene expression by binding to the 3’ends of specific mRNAs where they cause their degradation or translational repression. lnc RNAs can regulate gene expression by affecting chromatin remodeling, controlling transcription and RNA processing. They also contain binding sites for miRNAs thereby regulating their intracellular concentrations. Acting as sponges, they compete with mRNAs for binding to the miRNAs thus enhancing gene expression. In addition, it is widely known the direct influence that STAT3 has on astrocytes, a main cellular component of the glial scar, by regulating GFAP, Cx43 and Aquaporin4 (AQP4) expression, therefore impacting both astrocyte reactivity as well as astrocyte migration, two key events surrounding the glial scar (Justicia et al., 2000; Wang H. et al., 2018).

Preclinical studies have shown that targeting miRNAs holds potential as a strategy for promoting regeneration. The studies described below illustrate the diverse roles of these molecules play in regulating axon-outgrowth and the intracellular signaling pathways involved (reviewed by Li P. et al., 2021). Using SCI as a model of CNS injury it was shown that overexpression of miRNA-125b (which is down-regulated following SCI) promoted axon regeneration via the JAK/STAT pathway in neurons. Moreover, an *in vitro* study using the neuronal cell line N2A, showed that elevated expression of lnc RNA matlat-1 promotes neurite outgrowth by activating the MAPK/ERK signaling pathway.

Using a different CNS injury model, optic nerve crush, it was shown that the ncRNA, miRNA-19a, is downregulated in adult RGCs and this correlates with up-regulation of PTEN and loss of intrinsic ability to regenerate. Expression of miRNA-19a in adult RGCs (rat and human) promoted regeneration by lowering PTEN levels thus stimulating the mTOR signaling pathway. In another study the authors demonstrate that expression of miRNA-135s represses the expression of the transcription factor KLF4, a potent intrinsic repressor of axon regeneration in the optic nerve thereby promoting regeneration following a nerve crush. Additionally, another ncRNA, miRNA-30b has been shown to bind to the 3’end of Sema-3A mRNA (a potent inhibitor of regeneration in RGCs) blocking its ability to bind to its receptors thus preventing growth cone collapse. miRNA-21-5p has been shown to regulate fibrosis- related genes and it’s knockdown attenuates the formation of a fibrotic scar following SCI, one of the main obstacles for axon regeneration (Li Y. et al., 2021). Thus, these molecules exhibit multiple activities. They can upregulate growth promoting signaling pathways such as JAK/STAT and MAPK/ERK and can down regulate molecules that inhibit these pathways, such as PTEN. Additionally, they can attenuate the inhibitory properties of the glial scar by regulating the genes involved in fibrosis and inactivating inhibitory molecules such as SEMA3A. They are therefore potential novel therapeutic targets for nerve regeneration.

In yet another study two different injury models [optic nerve crush (ONC) and a DRG conditional injury models], were used to study the effects of Lin28a/b. Lin 28 regulates the expression of several genes, including the insulin-phosphati-dylinositol 3-kinase (PI3K). Thus, it has a direct effect on mammalian target of rapamycin (mTOR) signaling. Lin 28 is an evolutionarily conserved and converts somatic cells to (induced pluripotent stem cells) iPSCs. It additionally plays vital roles in determining body size and controls the onset of puberty. It also influences glucose metabolism, tissue regeneration and results in Akt activation and GSK3β inactivation (Wang X. W. et al., 2018). Its function with relation to axonal regeneration is via modulation of let-7 miRNA post-transcriptional processing resulting in a block on the production of mature let-7 miRNA. Let-7 miRNA negatively impacted regeneration on a *C. elegans* model (Morris et al., 2013), as well as impacting negatively peripheral axonal regeneration in a rat sciatic nerve injury model (Li et al., 2015). This led to the researchers to investigate the effect of Lin28 overexpression the aforementioned injury models but using mice.

They found that Lin28a or b overexpression was sufficient to induce sensory axon regeneration *in vivo* l in the DRG conditional lesion model. Curiously knock down of either Lin28a or b resulted in no change in regenerative capacity yet knockdown of both resulted in significant inhibition of axonal regeneration (Wang X. W. et al., 2018). The authors of this study suggested that their results were consistent with let-7 miRNA acting downstream of Lin28. They then demonstrated a similar positive effect of Lin28 on CNS regeneration using a mouse model of ONC. This resulted in robust optic nerve extension after injury. The effect was further enhanced when a combination approach was used. When lin28a overexpression was combined with PTEN knockdown long distance (750–2,000 μm) regeneration of optic nerve was observed. The two interventions acted synergistically as regeneration occurred more rapidly and was more robust than that observed with Lin28 overexpression alone. Moreover, axonal misguidance and growth cone bulb retractions were also reduced. Lin28’s effects on regeneration, in both the CNS and PNS were consistent with its actions on two pathways known to regulate axon outgrowth. The PI3K-Akt pathway is activated and the GSK3β pathway inactivated. Activation of Akt should result in enhanced axon-outgrowth via mTOR, and inactivation of Gsk-3β should result in inactivation of MAP1B and activation of APC and CRMP-2, all of which act to enhance axon-outgrowth (Figure 5). Recent studies have followed up on this seemingly generalized positive effect of Lin28 on axonal regeneration, using ONC and an SCI models, further confirming this positive regenerative outcome in yet another injury model (Nathan et al., 2020), something rather promising when considering the complexity and heterogeneity of regeneration in the CNS.

#### Microglial phenotype manipulation

In another study miRNA-124 secreted in small extracellular vesicles by anti-inflammatory M2 microglia was shown to reduce glial scar formation following ischemic stroke in mice. The study showed that these vesicles caused a reduction in astrocytic STAT3 and pSTAT3 (regulators of astrogliosis) via interaction with miRNA-124. GFAP which is downstream of STAT3 was also reduced. Encouragingly, these changes were accompanied by improvements in neurological function (Li Z. et al., 2021).

The effect of microglia phenotype in attaining successful axon regeneration is also highlighted in another study. Neonatal mice were subjected to a nerve crush of the spinal cord. Successful regeneration occurred across a scar-free lesion site. This was not replicated when older mice were used or if neonatal microglia are deleted confirming a central role for microglia in the process. The authors show that, in contrast to adult microglia, neonatal microglia undergo transient activation then return to a state of homeostasis. They secrete fibronectin and its binding proteins which form a bridge across the lesion site. It is of interest to note here that the Anderson paper identified fibronectin as one of the substrates favoring axon-outgrowth across the lesion site (Anderson et al., 2018). Neonatal microglia were also found to secrete both intracellular and extracellular proteinase inhibitors. When proteinase inhibitors were co-injected with neonatal microglia into an adult lesion site then regeneration was promoted (Li Y. et al., 2021). These results are very encouraging however, it is of note here that it is likely that the presence/levels of CSPGs could significantly influence the outcome since it has been shown that even neonatal rat microglia convert from M2-like wound resolving to M1-like inflammatory phenotype in the presence of CSPGs. This would be predicted to delay wound resolution resulting in the wound remaining in a chronic state (Francos-Quijorna et al., 2022).

#### Targeting PTEN

PTEN knockdown via shRNA, as discussed above, is effective at promoting regeneration of the CST. Moreover, PTEN knockdown is still effective at promoting growth of CST axons 1 year post-injury (T8 crush) and thus may be a potential treatment for chronic SCI (Du et al., 2015).

A further adaptation of this intervention came in the form of using rAAV2-retro to deliver an ShRNA against PTEN. Steward et al. (2020) were able to demonstrate that it is possible to target multiple pathways affected by SCI following a single injection of the vector at different levels of the spinal cord. These experiments, conducted in mice, resulted in significant regeneration of these pathways. The results from a follow up study from the same group support the idea that the outcome may be further improved by switching off shRNA PTEN expression once regeneration has been achieved. This is because long-term PTEN knockdown may have detrimental effects. Long-term mTOR expression results in *de novo* cell growth and abrogates activity-dependent immediate early gene induction and therefore may have adverse side effects, including preventing the formation of appropriate synapses required for functional recovery (Metcalfe et al., 2022). Another approach to achieve PTEN knockdown was used by Bhowmick and Abdul-Muneer (2021) who demonstrated that using peptides that block PTEN function, it is possible to stimulate corticospinal and raphespinal axon regeneration following SCI (hemi section). Although this injury is less severe than a contusion injury, the results are encouraging and because the use of such peptides doesn’t result in permanent PTEN-depletion it may confer an advantage over the constitutive expression of an shRNA against PTEN.

### Cell conversion approaches

Recent and novel research efforts have focused on direct cell reprograming to enhance the regenerative capacity on the CNS. One example is an experiment where reactive glia was targeted *in vivo* for cell conversion directly to functional neurons. This was accomplished by retroviral delivery of NeuroD1, a pro-neural transcription factor important for embryonic brain development and for adult neurogenesis. The astrocyte-related GFAP promoter was used to drive expression of NeuroD1 (GFAP:NeuroD1-IRES-GFP). The retroviral injection itself served as the stab injury. In this study the authors demonstrated that they were able to convert GFAP expressing cells into functional glutamatergic neurons (VGlut1 positive) expressing both NeuN and Tuj1 (commonly used neuronal markers). They then proceeded to convert NG2 + glia into neurons and succeeded in generating both glutamatergic and GABAergic (GAD67 positive) neurons. They then moved on to try and convert glial cells into fully functional neurons in a more challenging model, a 5xFAD Alzheimer’s disease aged mouse model. Not only they were again successful in cell conversion but also demonstrated synaptic and dendritic activity (SV2 and MAP2 positive respectively) as well as NMDA, Ca^2+^, K^+^ and glutamatergic and GABAergic currents (Guo et al., 2014).

In a ground-breaking follow up study, Zhang et al. (2020) utilized this cell conversion approach and took it to the next step: using this as means to restore neuronal tissue in the damaged CNS. This time, they decided to go with an AAV carrying NeuroD1 targeting astrocytes alone with the Cre-Flex system. Briefly, they were able to reduce the size of the injury (motor cortex stab wound) though this cell conversion therapy, seemingly eliciting some regenerative CNS capacity by converting astrocytes into neuronal tissue. They also observed that the pro-inflammatory events occurring in response to injury of the CNS were reduced and that the conversion also resulted in restoration of blood vessels and the blood brain barrier. It is of note that they saw no depletion in astrocytic numbers, suggesting a resident repopulation of astrocytes occurring alongside astrocyte-to-neuron conversion (Zhang et al., 2020). Although the authors didn’t particularly focus on this point, it is intriguing that this conversion event becomes more efficient when ChABC is injected with immature astrocytes which resulted in enhanced regenerative capacity (Filous et al., 2010; Filous and Silver, 2016). Another gap in this study is that aspects of functional recovery were not assessed. It is imperative to confirm that this neuronal tissue converted from astrocytes is indeed functionally replacing the damaged tissue. This could be achieved by using injury models that are compatible with experiments designed to measure behavioral recovery and/or restoration of synaptic plasticity and/or transmission.

### Stem cell and stem cell-like interventions

We have previously highlighted the importance of the stem-cell like state of glia in the efforts to promote central neuronal regeneration. This stems from the fact that a lot of these pluripotent cell states, particularly glia, are important for driving axonal guidance during the early developmental stages. It is true, for example, with respect to astrocytes, that at early phases of neurodevelopment and even in later infant life, they are crucial to perform functions such as axon guidance and regulation of synaptic fate of developing neurons at more immature developmental stages (Filous et al., 2010; Thompson et al., 2017). Little is known about the specific molecular mechanisms through which immature astrocytes guide axons, but it is thought that during development they have a better ability to integrate axonal guidance and repulsive signals than mature astrocytes (Filous and Silver, 2016). It is known, however, that these astrocytes perform essential functions during development. These functions include synapse formation (via cholesterol that is converted to steroid hormones acting on synaptic signaling) and maturation (via TNFα and NF-κB). These studies shed light on the roles that these glial cells play at early stages of development, having implications for synaptogenesis and potentially for synapse restoration.

Filous et al. (2010) employed a combination approach to investigate the efficacy of cell transplantation (immature astrocytes) with enzymatic digestion of CSPGs with ChABC. The rationale behind this approach was that the immature astrocytes would guide axonal processes across the glial scar and that ChABC would facilitate this process by degrading the dense ECM. Immature astrocytes are thought to guide axons in an MMP-2-dependent manner. Although mature astrocytes also secrete MMP-2 they do so in much smaller quantities which is the reason behind using immature astrocytes for transplantation. The outcome of this study was encouraging. They demonstrated that using this combination strategy, immature astrocytes were able to not only pass through a dense CSPG rim in culture (consisting of aggrecan/laminin) but also that they were able to cross and guide axonal processes directly through the glial scar produced in response to a micro-injury. Regenerating axons crossed the lesion site via astrocytic bridges and regenerating fibers extended from cingulum to the corpus callosum, a highly myelinated region of the brain (Filous et al., 2010). Interestingly, transplantation of immature astrocytes alone did not have a significant impact on regeneration. These findings add support to the idea that any successful approach to improve the regenerative process will involve a multitargeted strategy, i.e., a combination of interventions (Zhao and Fawcett, 2013; Filous and Silver, 2016; Adams and Gallo, 2018; Hu et al., 2018; Griffin et al., 2020). The findings also add to the growing list of combination therapies where ChABC enhances the effects of other interventions (Burnside et al., 2018).

Astrocytes either in immature state or in combination with neural stem cells (NSC) have been used with great success. In one study neonatal (immature state) astrocytes were transplanted into a rat SCI lesion using collagen as a scaffold. The authors demonstrated that this resulted in improved spinal cord repair (anatomical) and that this was accompanied by some functional improvements as evidenced by locomotor improvement (Joosten et al., 2004). Another example of using astrocytes as a tool to promote CNS regeneration is a study using a rat model of stroke where astrocytes were co-transplanted with NSCs. It was found that the astrocytes improved NSC survival, differentiation and proliferation (Luo et al., 2017). This suggests that NSC transplantation is likely to be more effective when the NSCs are co-injected with astrocytes. Multiple other studies have been published showing regenerative improvements after astrocyte or immature astrocyte injection directly to the injury site (Davies et al., 2006, 2011). Intriguingly, it has been shown in several cases that the beneficial effects of NSC transplantation are mediated through astrocytic-dependent mechanisms. An example of such as case is a study involving NSC transplantation in a mouse model of stoke (Bacigaluppi et al., 2016). Here they saw that treated mice had reduced corticospinal tract degeneration, increased axonal sprouting and dendritic arborization which resulted in long term functional recovery evaluated by the modified Neurological Severity Score and electrophysiology (i.e., synaptic plasticity) (Bacigaluppi et al., 2016). This positive outcome was seen to have occurred through the ability of NSC to promote upregulation of the glial glutamate transporter 1 (GLT-1) on astrocytes. This resulted in a reduction of peri-ischemic extracellular glutamate (Bacigaluppi et al., 2016). The described studies highlight the benefits of NSC transplantation as a method to promote CNS repair and also reveal the therapeutic potential of astrocytes and their roles in CNS regeneration, synaptic formation and maturation.

As discussed earlier, the superior regenerative capacity of PNS neurons if largely due to the functions and properties of SCs. This inspired many studies using SC transplantation as a method to enhance regeneration in the CNS, mostly using SCI as an injury model and their potential for clinical applications has been accepted (Bunge and Wood, 2012; Kanno et al., 2015). Recently an interesting study demonstrated the positive impact and outcome of transplanting SC precursors into the spinal cord in a rat model of SCI. The authors reported that the transplantation resulted in improvements in locomotor function which were highly significant when compared to the control group. It is of interest that the levels of locomotor recovery in the SC-transplanted group far exceeded the levels of recovery reported for animals receiving a mesenchymal stem cell transplant (De La Garza-Castro et al., 2018). These findings highlight not just the potential of SC transplantation but further emphasize the potential of stem cell directed strategies as well as the importance of stem cell origin and nature for determining regenerative outcomes. Studying the events surrounding neurodevelopment and/or where synaptogenesis and neurogenesis are successful will aid the development of novel axonal regenerative interventions.

## The need to build, cross and walk the bridge

The glial scar represents a major barrier to regenerating axons as previously mentioned. Therefore, it seems clear that for a regenerative process to occur, we need to build an axonal guidance pool involving a combination of interventions to create the basis for the physical and molecular bridges required to move beyond this territory and to build on this foundation a means of promoting the re-stabilization of synaptic plasticity and function.

While representing a considerable challenge, significant advances have been made in developing therapies that promote regeneration following CNS injury. The results from the studies documented above, give insights into which approaches will likely be needed to achieve successful regeneration. Although preclinical studies have given promising results, to date, no single intervention for SCI, stroke or traumatic brain injury has progressed from phase III clinical trials to gain MRHA/FDA approval. Thus, successful outcomes will likely require a combination of interventions. The studies described above have identified the major barriers to regeneration and potential therapeutic targets. The collective results suggest that an efficacious combined therapy will include at least four components: (1) a strategy to boost the intrinsic ability of a nerve cell to regenerate; (2) a strategy to counteract the inhibitory components of the glial scar; (3) a chemotactic cue to guide regenerating neurons to their correct synaptic targets; (4) include a form of rehabilitation. The studies described above suggest that the JAK/STAT and mTOR signaling pathways are promising targets for component 1. Component 2 could be achieved in several ways: (A) removal of CSPGs with ChABC and neutralization of myelin inhibitors with Nogo antibody; (B) blocking the downstream signaling pathways that mediate their action such as RhoA and GSK-3β, both of which can be blocked with Ibuprofen; (C) upregulating the production of regenerative substrates. An alternative approach is to provide a permissive surface across the lesion site via cellular transplants of NSC and/or immature astrocytes to create cellular bridges. Component 3 could be achieved by the delivery of neuron specific growth factors. This component will likely need to be tailored to the neuronal population in question. For instance, GDNF is chemotactic for propriospinal neurons (Anderson et al., 2018) whilst NT3 and CTNF may be more appropriate chemotactic signals to promote regeneration of CST neurons following SCI, stroke and onset of motor neuron disease (Richter and Roskams, 2009). For component 4, treadmill training, hand grasp training, electrical stimulation, intermittent hypoxia have been shown to improve functional outcomes of combination therapies (Muir et al., 2019), while rehabilitation for the case of degeneration seems more challenging to achieve.

With regard to the different alternatives for achieving component 2, a major strategy is using a degrading molecules of CSPG such as ChABC, either directly or in a viral vector transgene delivery. It is of note that preclinical studies have shown that ChABC exhibits synergy in almost all combination therapies tested and its actions do not overlap with any of them making it a desirable component of any therapy (Muir et al., 2019). ChABC also targets both intrinsic and extrinsic components, namely PTEN and RhoA knockdown and CSPG removal. Moreover, strategies that remove components of the ECM have the additional advantage of counteracting the non-specific inhibitory effects of these molecules such as trapping chemo-repulsive cues and impeding the binding of regenerative molecules such as integrins which are essential for axon outgrowth (Muir et al., 2019). Additionally, some of the CSPG-digestion products themselves have been shown to promote CNS regeneration (Rolls et al., 2004).

Future research will be needed to determine the optimal timing, dose and duration of each intervention. ChABC can be used as an example of the importance of determining these parameters. (1) Timing: when ChABC is delivered before or at the time of SCI, the outcome is detrimental, most likely because CSPGs are beneficial shortly after injury (Rolls et al., 2009; Sofroniew, 2015). The best outcomes were observed when ChABC delivery was initiated 48 h to 1 week post-injury (Muir et al., 2019). (2) Dose: when ChABC is given via injection, high doses were more efficacious than low doses (Muir et al., 2019). (3) Duration of delivery: delivery for 4 weeks following SCI resulted in significant improvements, but continued delivery for 8 weeks produced the best outcomes (Burnside et al., 2018).

Moreover, the timing of the application of each component is also likely to be critical. In fact, when rehabilitation was initiated 1 day post-injury (SCI) and ChABC at 4 days post-injury, no improvements were observed but in a different study where ChABC was delivered acutely and rehabilitation initiated 1 week post-injury, significant improvements in hand function were reported (Muir et al., 2019). These results suggest that optimal outcomes may be obtained by first stimulating plasticity (ChABC) then driving functional connections with rehabilitation. With regard to the timing of the different interventions, as in Anderson study (Anderson et al., 2018), boosting the ability of neurons to regenerate prior to SCI is not feasible in a clinical setting. Results obtained so far have identified several promising candidates for future combination therapies for CNS injuries. What is becoming apparent is a requirement for a method to tightly control the delivery timing of the different molecular components. A strategy would be using a gene switch similar to the one described in Burnside et al. (2018), that briefly uses a novel vector approach where in addition to using a doxycycline dependent gene regulatory system, they use a chimeric transactivator allowing the virus to avoid T-cell recognition (Burnside et al., 2018).

The vast number of events which follow CNS injury emphasize the relevance of using a multi- pronged approach for achieving successful CNS regeneration. In terms of the constituents of such a multicomponent therapy, several candidates stand out as having the greatest potential. The potential of SC or SC-like cell, or more specifically glia, is well documented and their importance for regeneration can be inferred by the fact that they take part in the regenerative process in species where the central regenerative capacity is intact (Kizil et al., 2012; Fontana et al., 2018; Lust and Tanaka, 2019) and during development/synaptogenesis (Orlando et al., 2012; Gallo and Deneen, 2014; Filous and Silver, 2016; Zhang et al., 2016). Moreover, their efficacy has been established by a wide variety of studies involving both human NSCs as well as immature glia transplanted alone or transplanted with NSCs (Filous et al., 2010; Xu et al., 2012; Faiz et al., 2015; Karova et al., 2019). Another component likely to be essential is ChABC, for the reasons given above, and growth factors as well as some form of rehabilitation. As emphasized earlier, the need for a multi-pronged strategy requires us to look at the mirage of events surrounding CNS injury, this includes neuroinflammation, intrinsic and extrinsic regenerative capacity, which in turn includes microtubule/actin dynamics, molecular pathways involved directly and indirectly and the effects of excitotoxicity on the therapeutical strategy employed.

Future studies should include a detailed characterization of the different cell types impacting the glial scar structure, of neural function, and particularly synaptic transmission and plasticity, and/or of animal behavior, which will constitute functional outcomes of the therapeutical strategies that will enable to distinguish between anatomical recovery alone from functional recovery. The assessment of each therapeutic intervention in several injury models is essential given the diversity of events following different injuries that depend on injury location, size, and type. The type of tissue damage that occurs following CNS injury is diverse, it can be due to degenerative disease, ischemia or traumatic injury. Besides the diversity associated to the pathology of these different types of tissue damage, any potential therapies will have one requirement in common: a method to enhance the regenerative capacity of the CNS. Hence, the potential impact of finding viable therapeutical strategies to improve central axonal regrowth after injury is of the utmost relevance. Targeting the inhibitory events preventing regeneration in the CNS, which might be epigenetic targets, cellular targets, or the pathways themselves may ultimately lead to a treatment (likely a combination of interventions) with far-reaching implications.

The present review described and discussed not only the key knowledge obtained from decades of research but also the recent advances in the field of CNS regeneration. Among this knowledge are the cellular and molecular processes that are known to occur following an injury as well as relevant points when designing therapeutical interventions. However, there are still important gaps in our knowledge that have compromised the development of a successful strategy for CNS regeneration. The main gap is the reduced information regarding the sequence of events following an injury in the CNS and its vast heterogeneity. Considering the state of the art of nerve regeneration presented in this review, we hope the near future research will identify and characterize the missing building blocks necessary to construct a bridge of knowledge that will allow the passage to the other side of the glial scar.

## Author contributions

GC, FR, and AS wrote the manuscript. GC and FR designed the illustrations. EM and SV wrote, designed, and revised the manuscript. All authors contributed to the article and approved the submitted version.
